# Protein Arginine Methylation: An Emerging Modification in Cancer Immunity and Immunotherapy

**DOI:** 10.3389/fimmu.2022.865964

**Published:** 2022-04-14

**Authors:** Weijing Dai, Jianguo Zhang, Siqi Li, Fajian He, Qiao Liu, Jun Gong, Zetian Yang, Yan Gong, Fang Tang, Zhihao Wang, Conghua Xie

**Affiliations:** ^1^ Department of Radiation and Medical Oncology, Zhongnan Hospital of Wuhan University, Wuhan, China; ^2^ Hubei Key Laboratory of Tumor Biological Behaviors, Zhongnan Hospital of Wuhan University, Wuhan, China; ^3^ Hubei Cancer Clinical Study Center, Zhongnan Hospital of Wuhan University, Wuhan, China; ^4^ Department of Thoracic Surgery, Zhongnan Hospital of Wuhan University, Wuhan, China; ^5^ Department of Biological Repositories, Zhongnan Hospital of Wuhan University, Wuhan, China; ^6^ Tumor Precision Diagnosis and Treatment Technology and Translational Medicine, Hubei Engineering Research Center, Zhongnan Hospital of Wuhan University, Wuhan, China

**Keywords:** protein arginine methyltransferases (PRMTs), cancer immunity, cancer immunotherapy, post translational - modification, molecular mechanism

## Abstract

In recent years, protein arginine methyltransferases (PRMTs) have emerged as new members of a gene expression regulator family in eukaryotes, and are associated with cancer pathogenesis and progression. Cancer immunotherapy has significantly improved cancer treatment in terms of overall survival and quality of life. Protein arginine methylation is an epigenetic modification function not only in transcription, RNA processing, and signal transduction cascades, but also in many cancer-immunity cycle processes. Arginine methylation is involved in the activation of anti-cancer immunity and the regulation of immunotherapy efficacy. In this review, we summarize the most up-to-date information on regulatory molecular mechanisms and different underlying arginine methylation signaling pathways in innate and adaptive immune responses during cancer. We also outline the potential of PRMT-inhibitors as effective combinatorial treatments with immunotherapy.

## 1 Introduction

Post-translational modification is a chemical modification that increases protein functional diversity *via* the covalent addition of a chemical group. The process modulates protein function and has emerged as a crucial regulator of multiple cellular processes in cancer. However, arginine methylation, unlike acetylation, ubiquitination, and phosphorylation, has received little attention until recently, predominantly due to the choppy nature of substrate detection and an absence of reliable antibodies and effective small-molecule inhibitors. Proteomics and inhibitor discovery have deepened our understanding of writers, readers, and regulators during arginine methylation. Protein arginine methyltransferases (PRMTs) catalyze arginine methylation as “writers”, and are mainly involved in transcription activation and repression, pre-mRNA splicing, and DNA damage responses (1). Arginine methylation also modulates the transcription of targeted loci, thereby mediating key cell processes to maintain tissue homeostasis and disease phenotypes. Because arginine methylation is a targetable modification, PRMT inhibitors have shown great potential as cancer therapies in preclinical models and clinical trials, to effectively improve patient survival (2). As immune system functions are well-established and vital for tumor surveillance and cancer treatment, rapidly emerging cancer immunotherapies are now posited as the fourth pillar of cancer treatment (alongside surgery, radiotherapy and chemotherapy) (3).

Cancer immunity typically eliminates tumor cells or limits their growth, but it also promotes tumor progression by establishing tumor microenvironments (TMEs), which adapt to tumor growth (4). Despite the effectiveness of immune checkpoint blockade (ICB) therapy, a minority of patients with advanced malignancies derive clinical benefits from these treatments, with the majority developing innate or acquired resistance (5). Therefore, further research into immunotherapy-based combination methods is required to improve overall survival in patients with advanced-stage cancer.

Given that cancer immune responses are continuous, dynamic, tightly-regulated, and well-designed processes, suppressive responses to immunotherapy may be attributed to several factors, including the TME immune phenotype and molecular mechanisms underlying intrinsic tumor resistance (6). Among issues relevant to optimal responses to immunotherapy are each step in a series of cascade amplification process called cancer-immunity cycle, interplay between innate and adaptive immunity as well as activation or loss of oncogenic signaling pathways. The classical cancer-immunity cycle includes antigen expression and presentation, T-cell trafficking and tumor infiltration, and the killing of target cells by cytotoxic T lymphocytes (CTLs). The innate immunity fully integrates, sustains, and amplifies this cycle. While epigenetic regulators are involved in the activation and translocation of immune cells to the TME, as well as subsequent immune responses, their specific roles in tumor immunity and their combined effects with immunotherapy remain unclear (7, 8).

Here, we review arginine methylation functions in tumor immunity from three perspectives: 1) cancer-immunity cycle involvement in antitumor responses, 2) Type I interferon (IFN) production and related signaling pathways, and 3) intrinsic tumor resistance mechanisms. Using these perspectives, we provide evidence highlighting the novel physiological roles of PRMTs in cancer immunity, and we ask if PRMT-inhibitors can be combined with immunotherapy to achieve maximum and long-lasting therapeutic effects in cancer.

## 2 PRMTs

### 2.1 PRMT Classification

**Figure 1:**
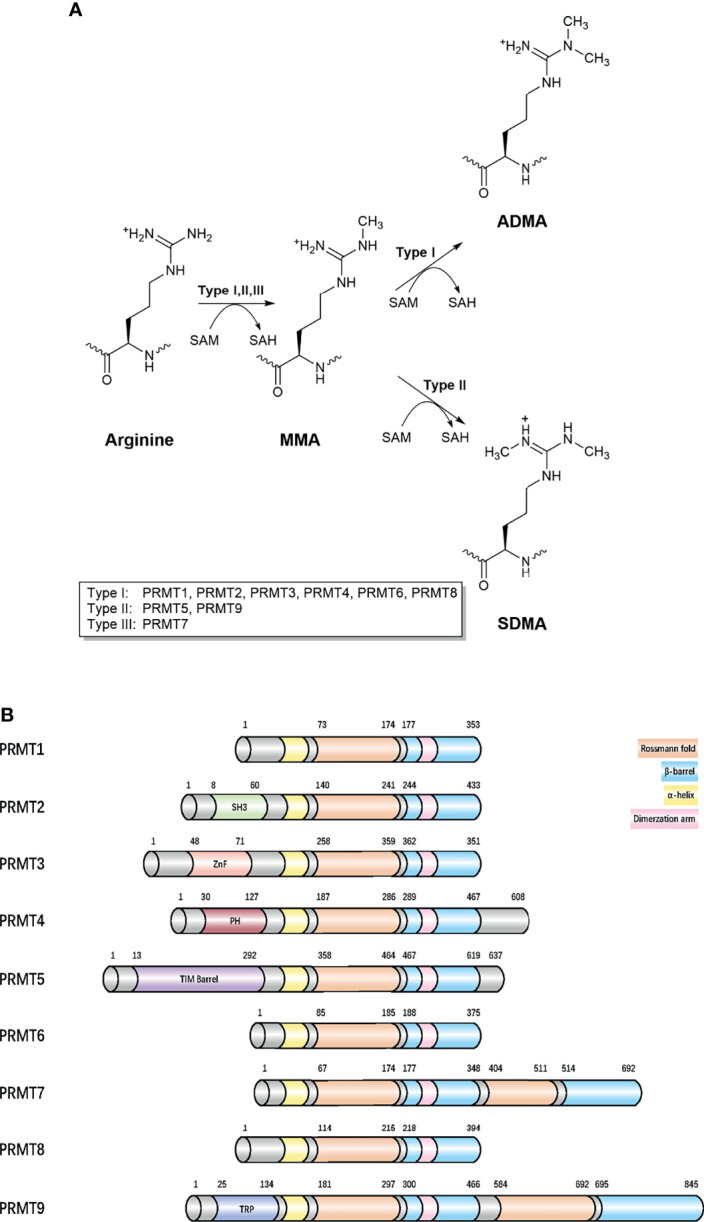
Classification of different methylarginines and protein arginine methyltransferases. **(A)** Arginine methylation is catalyzed by nine PRMTs. Type I, II, and III PRMTs generate monomethyl-arginine (Rme1, MMA) from arginine at first. Followed by asymmetrical dimethyl-arginine (Rme2a, aDMA) catalyzed by type I (PRMT1 PRMT2, PRMT3, CARM1, PRMT6, and PRMT8) and symmetrical dimethyl-arginine (Rme2s, sDMA) catalyzed by type II (PRMT5 and PRMT9). They added methyl groups to the nitrogen atoms of the guanidino group using *S*-adenosyl-L-methionine (AdoMet), converting it to *S*-adenosyl-L-homocysteine (AdoHcy). The type III, PRMT7 generates only monomethyl-arginine. **(B)** Domain architecture of human PRMTs. For all proteins, residue numbers are based on human protein sequences. The second Rossmann fold and β-barrel domain in the C-terminal region of PRMT7 are less similar with template sequences and catalytically inactive. The protein modules, such as SH3, ZnF, PH, and TPR can read arginine methylation marks. Protein sequences were found in UniProt. SH3, SH3 domain; ZnF, zinc finger motif; PH, Pleckstrin homology domain; TPR, tetratricopeptide repeat.

Arginine methylation mostly refers to the addition of methyl groups from S-adenosylmethionine (SAM) to the guanidino groups of arginine side chain, which is catalyzed by PRMTs (9). Five potential hydrogen bond donors are located in the guanidino group, with arginine residue methylation removing a potential hydrogen bond donor, thereby altering the shape of arginine residue without neutralizing cationic charges and generating physiological effects in most cells (10, 11). Currently, nine PRMTs (PRMT 1–9) have been identified in mammalian cells and are divided into three distinct groups according to catalytic activity: 1) ω-N^G^-monomethylarginine (MMA) refers to a single methyl group added to the terminal nitrogen atom, 2) asymmetric ω-N^G^-dimethylarginine (aDMA) refers to another methyl group placed on the same guanidino group based on MMA, and if placed on the other terminal guanidino nitrogen, this refers to 3) symmetric ω-N^G^, N’G-dimethylarginine (sDMA). Type I enzymes (PRMT1, PRMT2, PRMT3, PRMT4 (CARM1), PRMT6, and PRMT8) catalyze aDMA (12), whereas type II enzymes (PRMT5 and PRMT9) catalyze sDMA (13), and type III enzymes (PRMT7) only catalyze MMA (**Figure 1**). PRMTs participate in many biological processes, including gene expression regulation, DNA damage responses, and cell signaling (**Supplementary Table 1**). PRMTs are predominant and essential for human cells, especially PRMT1, which is responsible for 85% of aDMA, and displays a dramatic loss of methylation when knocked out (14). Moreover, it is speculated much crosstalk exists between PRMTs (15). PRMT1 loss causes the release of diverse substrates because they are no longer blocked by an aDMA modification, making these substrates switch to Type II and III PRMT targets (15). However, PRMTs have mutually exclusive functions even if they apply to a variety of identical substrates *in vitro* (16).

### 2.2 PRMT Functions in Cancer Biology

Previous studies reported that the elevated expression of PRMTs was correlated with tumorigenesis and poor prognosis in several cancers (17–19) (**Supplementary Table 2**). To date, most PRMTs are implicated in the regulation of cell processes associated with cancer maintenance, including epigenetic-mediated gene expression, mRNA splicing, and DNA damage responses (20). Among these pathways, epigenetic regulation is a prominent mechanism affecting cell activity, mainly due to PRMT depositing activation (histone H4R3me2a, H3R2me2s, H3R17me2a, and H3R26me2a) or repressive (H3R2me2a, H3R8me2a, H3R8me2s, and H4R3me2s) histone marks, which promote or suppress (respectively) key tumor-related genes (21). Early studies on nonhistone methylation identified roles for PRMTs in constitutive and alternative splicing regulation (22, 23). Indeed, PRMT5 and type I PRMT inhibition suppressed sDMA and aDMA functions in RNA-binding proteins involved in splicing regulation, leading to tumor-suppression responses in animal leukemia models with splicing factor mutations (24). The down-regulation of DNA damage repair has long been recognized as increasing genome instability to generate uncontrolled cancer development (25). Given that the arginine methylation of DNA damage repair related proteins, including MRE11, 53BP1, and hnRNPUL1, is required for DNA repair by homologous recombination repair and non-homologous end joining, PRMT inhibitors have been combined with chemotherapy to overcome resistance to cytotoxic treatments (26–28).

These encouraging findings suggest potent and exciting roles for PRMTs in cancer treatment. Another unexpected finding was the synergistic effect of PRMT inhibition in combination with immune checkpoint inhibitors (ICIs) (19). Therefore, studies clarifying the molecular mechanisms underlying PRMT depletion or inhibition could help identify novel “druggable” targets for immunotherapy.

## 3 PRMTs in Cancer Immunity

Recently, immuno-oncology has become a prominent area in cancer treatment, with ICIs identified as promising mono-therapies or combination therapies to treat different cancers (29). The stepwise events involved in the induction and execution of anticancer immune responses, so called cancer-immunity cycle, provide a theoretical basis for tumor immunotherapy (30). Within this context, it is important to understand the interplay between PRMTs and components in the cancer-immunity cycle, as the targeting of one or more cycle steps could enhance ongoing antitumor immune responses and/or stimulate new ones. Many studies have now shown that type I interferon (IFN) is critical for anticancer immune responses as IFN is a major regulator and mediator at each step of the cancer-immunity cycle (31, 32). Therefore, developing safe and efficient agonists to stimulate type I IFN could represent promising strategies for cancer immunotherapy. Indeed, intrinsic tumor defects are emerging as key molecular factors dictating the immune context of different cancer types, with impeding roles in local anti-tumor immune responses (33). The exploitation of relationships between intrinsic tumor genetic events and PRMTs is required to maximize inhibitor efficacy and develop personalized immune intervention strategies. Collectively, identifying methylation events in the cancer-immunity cycle, characterizing type I IFN production, and unravelling oncogenic pathways will provide comprehensive and profound insights on PRMTs in immuno-oncology and help identify new targets.

### 3.1 The Role of PRMTs in the Cancer-Immunity Cycle

**Figure 2:**
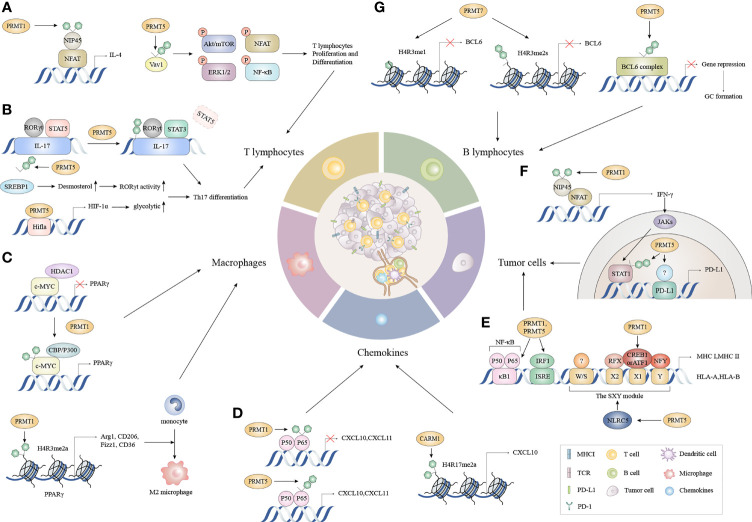
The functions of PRMTs in the cancer immune cycle. **(A)** During T lymphocyte proliferation and differentiation, the methylation of NFAT interacting protein NIP45 and the subsequent binding of PRMT1 to the NFAT transcriptional activation complex amplifies IL-4 production. Moreover, PRMT5 partially controls T cell signaling events, including AKT/mTOR, ERK1/2, NFAT, and NF-κB signaling by asymmetrically dimethylating Vav1 and promoting T cell proliferation and IL-2 production. **(B)** PRMT1 recruits RORγt to the IL-17 promoter and asymmetrically dimethylates histone 4 arginine 3 (H4R3me2a) to stabilize STAT3 activated by IL-6 rather than STAT5 activated by IL-2. This alleviates the inhibitory effects of STAT5 on the IL-17 promoter and promotes Th17 differentiation. Concomitantly, PRMT5 methylates the cholesterol biosynthesis regulator SREBP1 in T cells, promoting cholesterol biosynthesis enzymes to produce RORγt agonists, thereby promoting RORγt activity and driving Th17 differentiation gene expression. Besides cholesterol biosynthesis, PRMT5 directly modulates RORγt activation and promotes the glycolytic pathway by up-regulating *Hif1a*, which encodes HIF-1α. **(C)** c-Myc methylation by PRMT1 increases its binding capacity for p300 instead of HDAC1, and enhances c-Myc-p300 complex recruitment to PPARγ which triggers M2 transcription. Additionally, PRMT1 generates asymmetrically dimethylated histone H4 Arg3 (H4R3me2a) at the PPARγ promoter to ultimately promote M2 differentiation in response to IL4-STAT6 signaling. **(D)** CARM1 recruits p65 to the CXCL10 promoter and catalyzes H3R17me2a. CXCL10 and CXCL11 expression is triggered when PRMT5 symmetrically dimethylates p65 which increases p65 affinity for DNA. Conversely, PRMT1 asymmetrically dimethylates p65 which negatively regulates p65-DNA binding and transcriptional activity. **(E)** PRMT1 and PRMT5 mediate p65 arginine methylation functions differently on CXCL10 and CXCL11 induction. **(F)** The methylation of the NFAT cofactor protein NIP45, catalyzed by PRMT1, augments IFN-γ production and is followed by enhanced PD-L1 expression *via* IFNγ/JAK/STAT1 signaling. PRMT5 up-regulates PD-L1 *via* H3R2me2s on the STAT1 promoter and binds to an unknown transcription factor on the PD-L1 promoter. **(G)** PRMT5 interacts with complexes containing BCL6, encouraging BCL6-mediated transcriptional repression and allowing access to GC. While PRMT7 induces H4R3me1 and H4R3me2s at the BCL6 promoter, it represses BCL6 expression and limits GC formation. NFAT, nuclear factor of activated T cells; AKT/mTOR, protein kinase B/mammalian target of rapamycin; ERK, extracellular regulated protein kinases; NF-κB, nuclear factor kappa-B; RORγt, retinoid-related orphan receptor γt; STAT3, signal transducer and activator of transcription 3; STAT5, signal transducer and activator of transcription 5; SREBP1, sterol-regulatory element binding proteins; HDAC1, histone deacetylase 1; PPARγ, peroxisome proliferator-activated receptor; CXCL10, C-X-C motif chemokine ligand 10; CXCL11, C-X-C motif chemokine ligand 11; NIP45, NFAT-interacting protein 45; GC, Germinal center.

Considerable evidence suggests that tumor immunity is not an isolated process, but requires a series of stepwise events called the “cancer-immunuty cycle”, or a self-amplifying process where each step influences tumor development and prognosis by enhancing positive regulatory signals or inhibiting negative regulatory signals (30, 34, 35). The complete removal of malignant tumors is theoretically achievable (30). In this model, the escape of tumor cells is accomplished by disrupting specific steps in the cancer-immune cycle. The tumor-immune cycle begins with the release of tumor-associated neoantigens from tumor cells, which are recognized by antigen-presenting cells such as dendritic cells (DCs), B cells, and macrophages, to form antigenic peptide-major histocompatibility (MHC) complexes. Mature DCs migrate from the tumor to secondary lymphoid organs (SLOs) to deliver antigenic peptide-MHC complexes to CD4^+^T and CD8^+^T cells (36). T cell receptors (TCRs) recognize these complexes and generate a first signal, followed by the up-regulation of the transmembrane protein CD40L on the surface of Th cells. And then CD40L binds to CD40 on the surface of DCs, which greatly increases B7 expression and subsequently binds more strongly to CD28 on the surface of Th cells. This dual signal initiates the co-stimulation of T cells and produces cytokines, leading to the activation of effector T cells against cancer-specific antigens. Activated effector T cells then travel to the tumor *via* the circulation and create tumor-infiltrating immune cells. Finally, TCRs on CD8+ T cells recognize the antigenic peptide-MHC-I complex and kills target cancer cells, with antigens released by dead cancer cells into the next cycle (30) The potential alterations regulated by arginine methylation in cancer-immunity cycle are shown (**Figure 2**).

#### 3.1.1 PRMTs and Tumor Antigen Presentation

Many cancers evade immune rejection by suppressing MHC class I (MHC-I) antigen processing and presentation. MHC-I has attracted considerable attention as it presents endogenous peptide antigens to CD8^+^ T cells, inhibits interference with tumor recognition, and reduces CD8^+^ T cell cytotoxicity, which are primary effector cell types for ICB success (4). MHC-I down-regulation was observed in 40%–90% of human tumors, which affects MHC : TCR interactions and reduces cancer cell recognition by CD8^+^ T cells. IFN regulatory factors (IRFs) and nucleotide-binding domain leucine-rich repeat containing (NLR) family CARD domain containing 5 (NLRC5) are frequently associated with MHC-I down-regulation (37–40). MHC class II (MHC-II) molecules are mainly expressed by specialized antigen-presenting cells (APC) and present exogenous peptide antigens primarily to CD4^+^ T cells (41). Either MHC-I or MHC-II down-regulation is linked to a poor cancer prognosis, impaired ICB responses, and reduced tumor rejection rates in mouse models (42–44).

A recent study reported that PRMT5 controlled MHC-I abundance and antigen presentation be affecting NLRC5 transcription (19). PRMT5 down-regulation or the pharmacological inhibition of PRMT5 activity increased the expression of NLRC5 and its target genes, which was implicated in antigen processing and presentation. NLRC5 is a transcriptional activator of MHC-I genes when paired with transcription factors interacting with MHC-I promoter regulatory elements, such as the SXY module (39, 45). PRMT5 knockdown also increased NLRC5 expression after IFN-γ treatment, and was accompanied by increased MHC-I surface expression (19). NLRC5 expression was inversely correlated with PRMT5 expression in cancer cells (46). Therefore, targeting PRMT5 could be a promising strategy to increase MHC-I abundance and promote antigen presentation. Besides PRMT5, several other PRMTs were shown to negatively regulate MHC-I levels. In monocytes, PRMT1 inhibition decreased cyclic adenosine monophosphate (cAMP) response element binding (CREB) enrichment at the CRE site on the HLA-B (Major Histocompatibility Complex, Class I, B) promoter to increase MHC-I levels (47). In PRMT7 knockdown or PRMT7 inhibitor-treated murine melanoma B16 cells, MHC-I gene regulators such as *Nlrc5* and its target genes are also up-regulated, similar to the MS023 (PRMT1 inhibitor) group (48).

PRMT1 was shown to methylate the class II transactivator (CIITA) which is a key regulator of *MHC-II* expression, and associated and cooperated with transcription factor binding to the *MHC-II* promoter (49). The arginine methylation of CIITA led to its subsequent degradation and the down-regulation of MHC-II mRNA and protein levels (49). A recent study reported that PRMT5 negatively regulated MHC-II expression in MYC-driven hepatocellular carcinoma (50). MHC-II was significantly increased on tumor-infiltrating CD45.1^+^ leukocytes and DCs when MYC-overexpressing transgenic mice were treated with the PRMT5 inhibitor, GSK3326595 (50). PRMT5 silencing in MYC-overexpressing hepatocellular carcinoma cells directly regulated MHC-II expression by decreasing the *in vitro* H3R8me2s and H4R3me2s enrichment on CIITA and CD74 promoters (50). Therefore, targeting PRMT5 using small molecular inhibitors could induce immune cell infiltration into liver tumors and promote antigen presentation through MHC-II up-regulation. However, another study showed that the PRMT5-mediated accumulation of symmetrically dimethylated histone, H3R2 (H3R2me2s) in response to IFN-γ stimulation and was accompanied by CIITA enrichment at the MHC-II promoter; PRMT5 activated MHC-II transcription in an activity-dependent manner (51). These differential effects of PRMT5 on MHC-II transcriptional processes may have been due to different models, therefore, further studies are required to clarify these issues. CARM1 also activated IFN-γ-stimulated MHC-II transcription induced by CIITA in an arginine methyltransferase activity-dependent manner. Moreover, CARM1 methylated CREB binding protein at R714, R742, and R768 positions, thereby facilitating an association with the MHC-II promoter, and synergistically activating MHC-II transcription by CIITA (52). Together, PRMTs influenced the CIITA-mediated induction of MHC II by IFN *via* different mechanisms.

#### 3.1.2 PRMTs Mediate Immune Cell Migration by Regulating the Chemokines, CXCL10 and CXCL11

To execute anti-tumor actions, T cells migrate through the circulatory system to the tumor following initiation in lymph nodes. The CXCL9, -10, -11/CXCR3 axis mainly regulates immune cell migration, differentiation, and activation. CXCR3 is a surface-expressed receptor for CXCL9, CXCL10, and CXCL11 chemokines on immune and cancer cells. Chemokines recruit CTL, natural killer (NK), NK-T cells, and macrophages, then facilitate infiltration into cancer tissue *via* a chemotactic gradient, eliciting directional migration in response to IFN-γ, which is synergistically augmented by tumor necrosis factor-α (TNF-α) (53). The subcutaneous injection of lethal or sub-lethal doses of melanoma B16 cells into CXCR3-/-mice establish a critical role for CXCR3 as a receptor for CXCL9, CXCL10, and CXCL11 during CTL migration (54). Tumor-derived chemokines are also responsible for recruiting Th2 cells, Tregs, and myeloid-derived suppressor cells (MDSCs) (53). Therefore, CXCL9, CXCL10, and CXCL11 have crucial, nonredundant, cell-autonomous roles mediating immune cell infiltration into tumors, and inducing effective anti-tumor immunity. In addition, zeste homolog 2 (EZH2)-mediated histone lysine methylation and DNA methyltransferase-1 (DNMT-1)-mediated DNA methylation suppressed the production of CXCL9 and CXCL10 respectively, and then inhibited T-cell tumor homing. Therefore, epigenetic modulator treatments can eliminate this suppression and ultimately enhance cancer treatment (55). These observations suggest that epigenetic reprogramming of tumors can regulate T cell recruitment and the clinical efficacy of tumor immunotherapy, and arginine methylation as a widespread and critical epigenetic modality, affects these processes.

The catalytic structural domain of CARM1 directly interacts with p65, which is located in the nuclear factor kappa-B (NF-κB) Rel-homology domain, and serves as an essential dimerization motif (56). In TNF-α-stimulated CARM1 knockdown cells, p65 recruitment to the CXCL10 promoter was significantly defective and H3R17 methylation on the promoter was undetectable, whereas CARM1 re-introduction completely rescued these responses, suggesting CARM1 was required for p65 recruitment to the CXCL10 promoter and H3R17 methylation (56). PRMT5 also regulated the p65 association with the CXCL10 promoter, because PRMT5 triggered dimethylation of R30 and R35 sites on p65 and the DNA binding and nuclear translocation of p65 (57). The TNF-α-induction of CXCL10 in endothelial cells required PRMT5-mediated p65 arginine methylation. According to other research by this team, PRMT5 induced R174 dimethylation on p65 to modify binding to the CXCL11 promoter (58). Consistently, the PRMT5 symmetric dimethylation of p65 at R30 increased its affinity for DNA and triggered NF-κB-induced gene expression (59). In contrast, attenuating PRMT5 activity by short hairpin RNA or the small molecular inhibitor, EPZ015666 in melanoma B16 cells, increased CCL5 and CXCL10 expression after stimulation (19). PRMT5 also methylated IFI16, attenuated its binding to intracellular double-stranded DNA (dsDNA), and therefore induced CCL5 and CXCL10 expression (19). Although interactions between PRMT1 and CXCL10 or CXCL11 have not been described, PRMT1 asymmetrically dimethylated the conserved R30 residue of p65 (60). The PRMT1 methylation site is located in the RxxRxRxxC motif of the p65 DNA-binding L1 loop (60). Methylated p65 has a poor affinity for NF-κB shared oligonucleotides; it negatively regulated p65 DNA binding and transcriptional activity, thereby preventing NF-κB recruitment to target gene promoters (60). Therefore, we hypothesize PRMT1 could regulate chemokines at the transcriptional level. PRMT1, CARM1, and PRMT5 all bound directly to the Rel homology domain of p65, owing to the fact the interaction was mediated by a central region containing the methyltransferase structural domain, which was evolutionarily conserved (61). Overall, PRMT-chemokine interactions are dynamic and context-dependent, such that symmetric and asymmetric dimethylation may offset and occur at various stages of NF-κB reactions, thereby recruiting distinct effector molecules and producing diverse biological effects. Chemokine production should be promoted to facilitate the recruitment of tumor-infiltrating lymphocytes by selectively targeting distinct PRMTs.

#### 3.1.3 Overcoming Inhibitory Networks in the TME

The TME is now viewed as a complex tumor ecosystem rather than a tumor cell-centered growth pattern (62). To better understand how the TME functions, we must understand its extracellular matrix and stromal cell composition. Stromal cells are categorized as follows; infiltrating immune cells (IIC), angiogenic vascular cells, and cancer-associated fibroblasts (63). Antigen-specific infiltrating immune cells comprise innate immune cell types (macrophages (Mφs), dendritic cells (DCs), MDSCs, NKs, mast cells, neutrophils, and adaptive immune cells (T and B lymphocytes) that respond to a variety of stimuli) (64). While initially recruited from surrounding tissue or bone marrow to envelop tumor tissue, these cells are eventually “re-educated” to become different cell types that generate chronic inflammation and angiogenesis. In this environment, cancer cells and surrounding stroma crosstalk with each other by suppressing immunologically beneficial genes, and inducing aberrant pathway signaling, ultimately producing potent cytokines and chemokines that influence host immune surveillance to sustain cancer cell growth, progression, and metastasis. Much clinical evidence now shows that the TME significantly impacts immunotherapy efficacy and clinical outcomes, and that PRMTs may provide new therapeutic targets for tumor immunotherapy by modulating the TME toward high lymphocyte infiltration.

##### 3.1.3.1 Targeting T Cell Proliferation and Differentiation

Because CD8-effective T cells are the most potent tumor-killing cells, T-cell populations are highly topical research themes. CD8^+^ cytotoxic T cells and CD4^+^ T helper cells make-up T cell subpopulations in the TME. Naive CD4^+^ cells mainly differentiate into four distinct populations: T helper 1 (Th1), Th2, Th17, and regulatory T (Treg) cells. Their differentiation is determined by signal patterns received during their initial interactions with antigens (65). T cells in the TME not only exert anti-tumor effector functions, but some promote tumor growth. Among these, CD8^+^ cytotoxic T cells and Th1 cells, commonly known as CTLs, have tumor-killing roles. The latter cells are characterized by the production of IFN-γ, TNF-α, and interleukin (IL)-2, which support the former cell-type or act as cytotoxic T cells to directly remove tumor cells (66). Th2 cells support B cells by releasing IL-4, IL-5, and IL-13, while Th17 cells promote tumor growth by producing IL-17, IL-21, and IL-22 (65, 67). Additionally, Tregs attenuate T-cell-mediated antitumor immune responses by directly inhibiting T-cell function and cytokine production, or indirectly inhibiting antigen presentation to hinder T-cell activation (68, 69). A high ratio of Tregs to CD8^+^ T cells usually indicates a poor cancer prognosis (70). CD8^+^ cytotoxic T cells frequently fail to inhibit tumor formation during tumorigenesis due to T-cell exhaustion, which is defined as the persistent expression of T-cell inhibitory receptors and the progressive loss of IFN-γ production and degranulation functions in response to long-term factors such as chronic inflammation or cancer stimulation (71). IL-12 and IFN-γ polarize naïve CD4^+^ T cell toward Th1 cells through actions of the signal transducer and activator of T box transcription factor T-bet. Th2 cell differentiation requires GATA3, which is downstream of IL-4. Th17 cell differentiation requires retinoid-related orphan receptor (ROR)γt which is a transcription factor induced by transforming growth factor-β(TGF-β) in combination with IL-6, IL-21, and IL-23. Treg cell differentiation also requires forkhead box P3 (FOXP3) as a transcriptional factor.

The comprehensive characterization of arginine methylation in primary T cells identified the regulatory roles of PRMTs in T cell fate decisions. Using mass spectrometry, Geoghegan et al. identified 2,502 arginine methylation sites in 1,257 proteins in human T cells (72). On the list were T cell antigen receptor signal machinery components and key transcription factors regulating T cell fate determination. Moreover, these authors quantified changes in arginine methylation occupancy during primary T cell differentiation, and demonstrated changes in arginine methylation stoichiometry during cell stimulation in a subset of proteins critical to T cell differentiation (72).

Three independent studies using T cell-specific PRMT5-deficient mice corroborated the key role of PRMT5 in maintaining peripheral T cells, and guiding the transition of naive T cells to an effector/memory phenotype (73–75). Inoue et al. reported that when compared with control mice, *PRMT5* T cell-specific homozygous-deficient mice had lower invariant NK (iNK) T, CD4^+^ T and CD8^+^ T cell numbers. Also, PRMT5 was required for T cell survival and proliferation (74). Tanaka et al. showed that PRMT5 was critical for T cell transition from a naïve to an activated phenotype, and also the maintenance of already mature T cells (73). Lindsay et al. also confirmed that PRMT5 was required to maintain normal iNKT, CD4^+^ T, and CD8^+^ peripheral T cell populations (75). Both the activity of symmetric arginine dimethylation and PRMT5 expression levels were significantly induced in activated T cells. Moreover, Inoue et al. also reported that PRMT5 facilitated pre-mRNA splicing from *Il2rg* and *Jak3* to increase their expression levels to maintain T cell populations. *Il2rg* encodes γc, which is a receptor subunit shared by cytokines, including IL-2, IL-4, IL-7, IL-9, IL-15, and IL-21 (76). The γc cytokine family tightly regulate lymphocyte development, and function *via* the tyrosine kinases JAK1 and JAK3 (77). Defective lymphocyte development, including T, B, NK, and iNKT cells is observed in mice deficient in either γc or JAK3. These authors also showed that the symmetric dimethylation of arginine residues in Sm proteins by PRMT5 possibly accounted for splicing changes caused by PRMT5 deficiency (74).

High density-CD8^+^ cytotoxic T cells are clearly associated with a better prognosis in many cancers (78). Therefore, correlations between PRMTs, CD8^+^ T cell proliferation, and cytokine production ability have been investigated in numerous studies. Kumar et al. showed that CARM1 inhibition increased tumor-infiltrating CD8^+^ T cell numbers, promoted granzyme B and IFN-γ expression, and Ki-67 marker proliferation, which facilitated a stronger tumor-killing capacity in CD8^+^ T cells, suggesting CARM1 inhibition enhanced CD8^+^ T proliferation and cytokine production (79). Kumar et al. also showed that Carm1-knockout T cells expressed higher levels of memory CD8^+^ T cell-associated transcription factors, such as *Tcf7* and *Myb* (79). *Myb* promoted memory CD8^+^ T cell formation *via* the transcriptional activation of *Tcf7* and *Zeb2* repression (80). Notably, Carm1-knockout T cells co-expressed low levels of the inhibitory receptors PD-1 and TIM3, which are T cell exhaustion markers (79). Distinct from CARM1, PRMT5 inhibition suppressed the induction of tumor antigen-specific CD8^+^ T cells *in vitro* (81). PRMT methylation reactions converted SAM, as a methyl-donating cofactor, into S-adenosyl-l-homocysteine (SAH), while SAM was also converted to methylthioadenosine (MTA). Both SAH and MTA inhibited PRMTs in feedback loops, while MTA effectively inhibited PRMT5 activity (82). MTA reduced CD8^+^ T cell proliferation and viability, as it inhibited effector functions in human antigen-specific CD8^+^ cytotoxic T cells by reducing the degranulation capacity of CD8^+^ T cells, and inhibiting IFN-γ and IL-2 secretion in a dose-dependent manner (81). Strobl et al. reported that the PRMT5-specific methyltransferase inhibitor, EPZ015666 suppressed CD8^+^ T cell proliferation, viability, and functionality (83). Additionally, these authors associated PRMT5 with the selective MDM4 splicing which induced the transduction of the p53 signaling cascade to mediate cell-cycle arrest and apoptosis in CD8+ T cells. PRMT5 inhibition also reduced protein kinase B (AKT)/mammalian target of rapamycin (mTOR) signaling, which impaired CD8^+^ T cell proliferation and survival (83).

CD28 bound to B7 on APCs, and co-stimulation with TCR reduced the activation threshold of T cells, thereby promoting CD4^+^ T cell differentiation and Th2-type cytokine expression (84). Blanchet et al. reported that CD28 co-stimulatory signals induced arginine methylation in T cells (85). Given that SAH was rapidly eliminated by S-adenosyl-L-homocysteine hydrolase (SAHase) and SAH inhibited PRMT activities, SAHase serves as PRMTs activator. Pharmacological SAHase blockers strongly inhibited the activation of several key proteins stimulated by TCR signaling, including AKT, extracellular regulated protein kinases (ERK)1/2, and NF-κB. leads to impaired CD4^+^ T cells activation (86). Therefore, PRMTs may have important roles in CD4^+^ T cell activation and differentiation. Selective inhibitors for PRMT5 could suppress Th2 cell proliferation (87). Webb et al. observed that in *PRMT5* T cell-specific homozygous-deficient mice, the proportion of IFN-γ^+^T-bet^+^, IFN-γ^+^, and T-bet^+^ T cells increased during Th1 cell differentiation, but total differentiated Th1 cell numbers were reduced, more than likely due to reduced proliferation of Th1 (75). Additionally, these authors observed that the percentage of GATA-3^+^IL-4^+^ and IL-4^+^ T cells increased during Th2 differentiation (75). So PRMT5 inhibition enhanced CD4^+^ T cell differentiation but suppressed proliferation. Mechanistically, SAHase inhibitors were shown to reduce the arginine methylation of Vav Guanine Nucleotide Exchange Factor 1 (Vav1), an essential molecule, leading to restricted CD4^+^ T cell activation (86, 88). Consistently, arginine methylated Vav1 accumulated in the nucleus, and activated the transcriptional complex nuclear factor of activated T cells (NFAT) and NF-κB to promote IL-2 production and T cell proliferation (85). PRMT5 also regulated IL-2 transcription by methylating particular sDMA-containing proteins (89). Moreover, reduced IL-2 secretion by PRMT5 inhibitors helped suppress Th1 cell proliferation and differentiation (87). PRMT1 also methylated the NFAT interacting protein, NIP45 which facilitated an association with NFAT, a necessary modification for the NIP45-induced potent augmentation of NFAT transactivation and PRMT1 co-activation of the IL-4 promoter (90, 91). Collectively, SAHase inhibitors partially controlled proteins stimulated by TCR signaling through reduced Vav1 methylation, thereby inhibiting CD4^+^ T cell activation. PRMT5 also promoted IL-2 production by methylating Vav1 or NF-45 and NF-90. Given that IL-2 has critical roles in the polarization of naive CD4^+^ T cells to the Th2 phenotype, and in T cell proliferation, the high IL-2 expression levels induced by PRMT5 contributed to CD4^+^ T cell differentiation and proliferation (92, 93).

Recent studies showed that arginine methylation dysregulation contributed to Th17 differentiation. On one hand, the pharmacological inhibition of PRMT1 prevented Th17 cell generation (94). Mechanistically, in response to IL-6 and transforming growth factor-β (TGF-β), PRMT1 interacted with a key transcription factor in Th17 cell differentiation, RORγt, and induced IL-17 transcription and expression, which characterized the Th17 phenotype (94). After combination with RORγt, PRMT1 was recruited to the IL-17 promoter and asymmetrically dimethylated histone 4 arginine 3 (H4R3). The methylation of H4R3 stabilized STAT3 activated by IL-6 and removed STAT5 activated by IL-2, thereby alleviating the inhibitory effect of STAT5 on the IL-17 promoter and encouraging Th17 differentiation (95). However, on the other hand, Th17 differentiation was severely impeded in a T cell-specific PRMT5 deficient model, with the number of RORγt^+^IL-17^+^, IL-17^+^, and RORγt^+^ Th17 cells were very low in this model (96). This situation may have arisen due to the following: PRMT5 symmetrically dimethylated SREBP1 at R321, which inhibited its phosphorylation at S430 and prevented SREBP1 degradation *via* the ubiquitin-proteasome pathway (96). This change promoted the stability and transcriptional activity of SREBP1, a gene transactivator that promotes cholesterol biosynthesis, resulting in the accumulation of a potent endogenous RORγ agonist, desmosterol (75, 97). Besides its effects on cholesterol biosynthesis, PRMT5 also enhanced the glycolytic machinery to regulate Th17 differentiation. PRMT5 inhibition suppressed the expression of the glycolysis-regulating factor, hypoxia-inducible factor 1-α (HIF-1α) to promote Th17 differentiation *via* the direct transcriptional activation of RORγt, and promotion of the glycolytic pathway (75, 83, 98). Overall, by methylating histones to activate the IL-17 promoter and regulate metabolic reprogramming, including glycolysis and lipid metabolism following T cell activation, PRMTs promoted Th17 differentiation.

For Treg cells, FOXP3 was reported as the fate-determining transcriptional regulator, with high FOXP3 levels and stability required for Treg cell immunosuppressive activity (99, 100). PRMTs actively regulated the differentiation and suppressive activity of Tregs by regulating FOXP3. The PRMT1-catalyzed direct methylation of FOXP3 at R48 and R51 enhanced the suppressive function of Treg cells (101). Similarly, PRMT1 may also have affected Treg activity by regulating other proteins interacting with FOXP3, such as Forkhead box protein O1 (FOXO1) and the transcription factor Runt-related transcription factor 1 (Runx1) (102, 103). PRMT1 methylated FOXO1 at R248 and R250 to stabilize the protein, and also methylated Runx1 at R206 and R21 to potentiate its transcriptional activity (104, 105). Besides, PRMT1 inhibitor AMI-1 treatment strengthened Tregs sensitivity to TGF-β that induced FOXP3 expression and increased the frequency and suppressive effect of Tregs via directly enhancing FOXP3 expression (106, 107). Apart from PRMT1, PRMT5 also regulated *FOXP3* expression in naïve T cells by influencing DNMT1 expression which controlled CpG methylation in the FOXP3 promoter, and therefore enhanced transcriptional accessibility to FOXP3 regulatory regions (108, 109). Additionally, PRMT5 inhibition affected H3K27me3 modification in the FOXP3 promoter, and facilitated *FOXP3* expression (106). As PRMT5 interacted with EZH2, culminating in increased H3K27me3 deposition, H3K27me3 alterations in PRMT5 deficient cells may have been induced by PRMT5-EZH2 interactions (110). In contrast, Yasyhiro et al., in a Treg cell-specific PRMT5-deficiency model, observed the suppressive activity and effects/memory phenotype of Tregs were reduced, resulting from the decreased symmetric dimethylation marker of FOXP3 R51 locus (111). However, these authors discovered later that PRMT5 deficiency was possibly linked to defective maintenance, activation, and proliferation of Treg cells *via* impaired γc signaling rather than methylated FOXP3 (73).

##### 3.1.3.2 Targeting B Cells Activation and Differentiation

While T cells have long been the focus of tumor immunity research, other immune cell subsets are relevant to this field, particularly with respect to the tertiary lymphoid structure (TLS) concept. Rather than requiring DCs to migrate from the tumor site to SLOs, the TLS hypothesis posits this phenomenon occurs directly in ectopic lymphoid organs, generated by non-lymphoid tissue infiltrating tumor margins and stroma (112). Such structures consist of a CD3^+^ T cell zone and a CD20^+^ B cell zone, with the former containing DCs positive for DC-lysosome-associated membrane glycoprotein and fibroblast reticulocytes, and the latter containing germinal centers, plasma cells, follicular DCs, and antigen-antibody complexes (113). In the TLS, tumor-associated antigens trigger B cell transformation into an effector/memory phenotype that produces antibodies, and presents tumor antigens to T cells, thereby boosting the prognostic function of CD8+ T cells and promoting T cell growth (113). Elevated CD8^+^ and CD4^+^ T cell levels co-localizing with considerable CD20^+^ B cell infiltration was associated with long-term clinical survival (114). Moreover, several chemokines were secreted by B cells to recruit DCs, T cells, and NK cells (113). Both clinical cohort and analytical studies of the TME identified positive correlations between patient responses to ICB therapy and B cell infiltration and TLS formation. These studies highlighted the hitherto unappreciated role of B cells in human anti-tumor immunity, therefore ICB-insensitive tumors may better respond to emerging therapies targeting B-cell activity (115–117).

PRMT1, PRMT5, and PRMT7 regulate pre-B cell proliferation and differentiation, germinal center formation, and antibody responses. In B-cell-specific PRMT1-deficient mice, B cell numbers in the bone marrow and other peripheral lymphoid organs were significantly reduced (118), while B cell development was severely curtailed at the pre-B cell stage (119). Activated B cells showed increased PRMT1 and arginine methylated proteins levels. By down-regulating the recombination activating genes, *Rag1* and *Rag2*, FOXO1 blocked the progression of B-cell differentiation beyond the pro-B cell stage (120). Yamagata et al. showed that PRMT1 directly methylated FOXO1 to inhibit phosphorylation by AKT, and subsequent proteasomal degradation (105). Moreover, PRMT1 also methylated the Igα subunit of B-cell receptor (BCR) to inhibit BCR-induced Syk activation and phosphoinositide 3-kinase (PI3K) signaling, blocked FOXO protein degradation, and promoted pre-B cell differentiation rather than proliferation (121, 122). Thus, PRMT1 negatively regulated B-cell signaling events such as class switch recombination and antigen selection *via* PI3K-FOXO1 signaling, thus facilitating development from pre-B cells to immature B cells. Additionally, PRMT1 mediated the arginine methylation of CDK4, destabilized the CDK4-Cyclin-D3 complex, induced cell-cycle arrest, and promoted pre-B cell differentiation (119). In a peripheral B-cell compartment PRMT1-deficient mouse model, mature B cell proliferation and differentiation after stimulation was severely hampered, germinal center B (GC-B) cell generation was defective, and memory B cell activation and proliferation were considerably reduced (121). PRMT1 also maintained follicular B (FO-B) cell numbers and promoted germinal center (GC) formation upon FO-B cell activation (118). PRMT1 protected activated B cells from apoptosis by regulating the expression of BCL2 family pro-survival proteins, MCL1, BCL2, A1, and BCLX (121).

Litzler et al. reported that both PRMT5 protein and sDMA levels were up-regulated in proliferating B cell stages, and that PRMT5 promoted the survival of activated B cells (123). PRMT5 was necessary for B cell development in Pro-B and Pre-B cells. PRMT5 protected mature B cells from apoptosis and promoted proliferation and GC formation by regulating B cell transcription and splicing fidelity (123). Moreover, PRMT5 directly interacted and methylated BCL6 to facilitate BCL6-mediated transcriptional repression, thus regulating GC formation (124). The BCL6 target genes *Irf4* and *Prdm1*, which mediated plasma cell differentiation, were also down-regulated by PRMT5 (123).

Using a B cell specific PRMT7 knockout mouse model, Ying et al. demonstrated that loss of PRMT7 impaired B cell development, decreased marginal zone B (MZ-B) cells, increased FO-B cells, and promoted GC formation after immunization (125). PRMT7 depletion increased *Bcl6* expression by recruiting H4R3me1 and symmetric H4R3me2 to the *Bcl6* promoter. The differentiation of IgA- and IgG1-secreting plasma cell subtypes was also decreased in PRMT7-deficient mice, probably because PRMT7 loss induced Bcl6 up-regulation and inhibited *Irf4* and *Prdm1* in GC-B cells (125).

##### 3.1.3.3 Targeting Tumor-Associated Macrophage (TAM) Differentiation and the Expression of Other Immune Cells

Mounting evidence has now indicated that the TME alters immune cells to suppress immune surveillance and immunological responses (126). Classically activated M1 macrophages tend to phagocytose tumor cells by establishing a pro-inflammatory environment, and facilitating Th1 and CTL responses. However, alternatively activated M2 macrophages such as TAMs promote tumor growth and invasion by boosting Th2 polarization, tumor angiogenesis, and inhibiting anti-tumor immune responses (127). DCs specialize in antigen processing and presentation, while NKs efficiently identify tumor cells and use perforin/granzyme-mediated cytotoxicity to limit tumor growth, while accumulated MDSCs exert prominent immunosuppressive effects rather than differentiation into mature myeloid cells (128).

Using a myeloid-specific PRMT1 knock-out mouse model, Tikhanovich et al. showed that PRMT1 was required for M2 macrophage differentiation. PRMT1 induction was observed during monocyte to macrophage differentiation, which led to H4R3me2a deposition on the peroxisome proliferator-activated receptor γ (PPARγ) promoter which enhanced PPARγ expression (a key transcription factor required for M2 phenotype differentiation). The macrophage-specific deletion of PPARγ impaired the maturation of alternatively activated M2 macrophages (129, 130). In line with this study, PRMT1 induced macrophage polarization to M2 types and increased IL-6 and downstream STAT3 expression by promoting PPARγ-dependent macrophage efferocytosis to consume apoptotic bodies in a mouse model of alcohol-dependent hepatocellular carcinoma (131). Also, PRMT1 deficiency decreased M2 types through the c-Myc-mediated PPARγ pathway, which influenced the trafficking, efferocytosis, phagocytosis, and alternative activation of macrophages (132–134). PRMT1 also methylated c-Myc at R299 and R346 to up-regulate its capacity for binding to p300, instead of HDAC1. This enhanced c-Myc-p300 complex recruitment to genes triggered subsequent M2 transcription, such as PPARγ and mannose receptor C-type 1(MRC1) promoters (129). Furthermore, PRMTs influenced c-MYC expression and function in other cell lines as follows: 1) PRMT1 and CARM1 deposited H4R3me2a markers on the c-MYC gene promoter, which were read by the Tudor domain-containing protein 3 (TDRD3)-topoisomerase IIIB (TOP3B) complex, and facilitated *c-MYC* transcription (135). 2) PRMT5 methylated the R218 and R225 sites in heterogeneous nuclear ribonucleoprotein A1 (hnRNP A1) to regulate the internal ribosome entry site (IRES)-dependent translation of c-MYC (136). 3) The enzyme-dependent inhibition of c-MYC polyubiquitination by PRMT3 eliminated ubiquitin-mediated C-MYC degradation (137), and 4) PRMT5-mediated H4R3me2 labeling was rich in c-Myc-binding to CANNTG E-box elements, and also regulated MYC function (138).

PRMT6 also promoted tumor progression by facilitating macrophage differentiation to the M2 phenotype rather than the M1 phenotype, and supporting tumor neoangiogenesis. PRMT6 interacted with ILF2 in lung adenocarcinoma to regulate the synthesis of macrophage migration inhibitory factor to induce alternate TAM activation (139, 140).

Arginine methylation also affected other immune cells, except macrophages. The CARM1 small molecule inhibitor, EZM2302 treated tumors were infiltrated by a large number of dendritic cells, cross-presenting cDC1 cells, and NK cells. Consistently, in a PRMT5-knockdown tumor model, a higher abundance of NKs, DCs, and MDSCs were observed when compared with the control-knockdown model (19, 79).

When combined, PRMTs have essential roles regulating macrophage differentiation and NK, DC, and MDSC cell proliferation. These functions not only provide novel perspectives for alternate macrophage activation, but they could identify new therapeutic targets for cancer.

### 3.2 PRMTs and Type I IFN Signaling

Type I IFNs facilitate cancer immunosurveillance, antitumor immunity, and immunotherapy efficacy in several ways, most notably by stimulating DC maturation and improving their ability to process and present antigens in the cancer-innate immunity cycle (141). Type IFNs also increase the expression of cytotoxic mediators in CTLs and prevent NK cells from purging activated CD8^+^ CTLs, while simultaneously maintaining the memory phenotype and influencing Treg suppressive functions (142). When different pattern recognition receptor (PRR) classes, including Toll-like receptors (TLRs), RNA-sensing retinoic acid-inducible gene (RIG)-I-like receptors (RLRs), and cytosolic DNA sensors recognize pathogen associated molecular patterns or damage associated molecular patterns, TBK1 recruitment, together with the successive activation of IRF3/7 and NF-κB is induced, which conventionally increase IFN-stimulated gene (ISG) expression and type I IFN generation (143). It is conceivable that the appropriate stimulation of Type I IFNs and related signaling pathways could be developed as effective anti-tumor immunotherapies.

#### 3.2.1 PRMT and TLR Pathways

**Figure 3:**
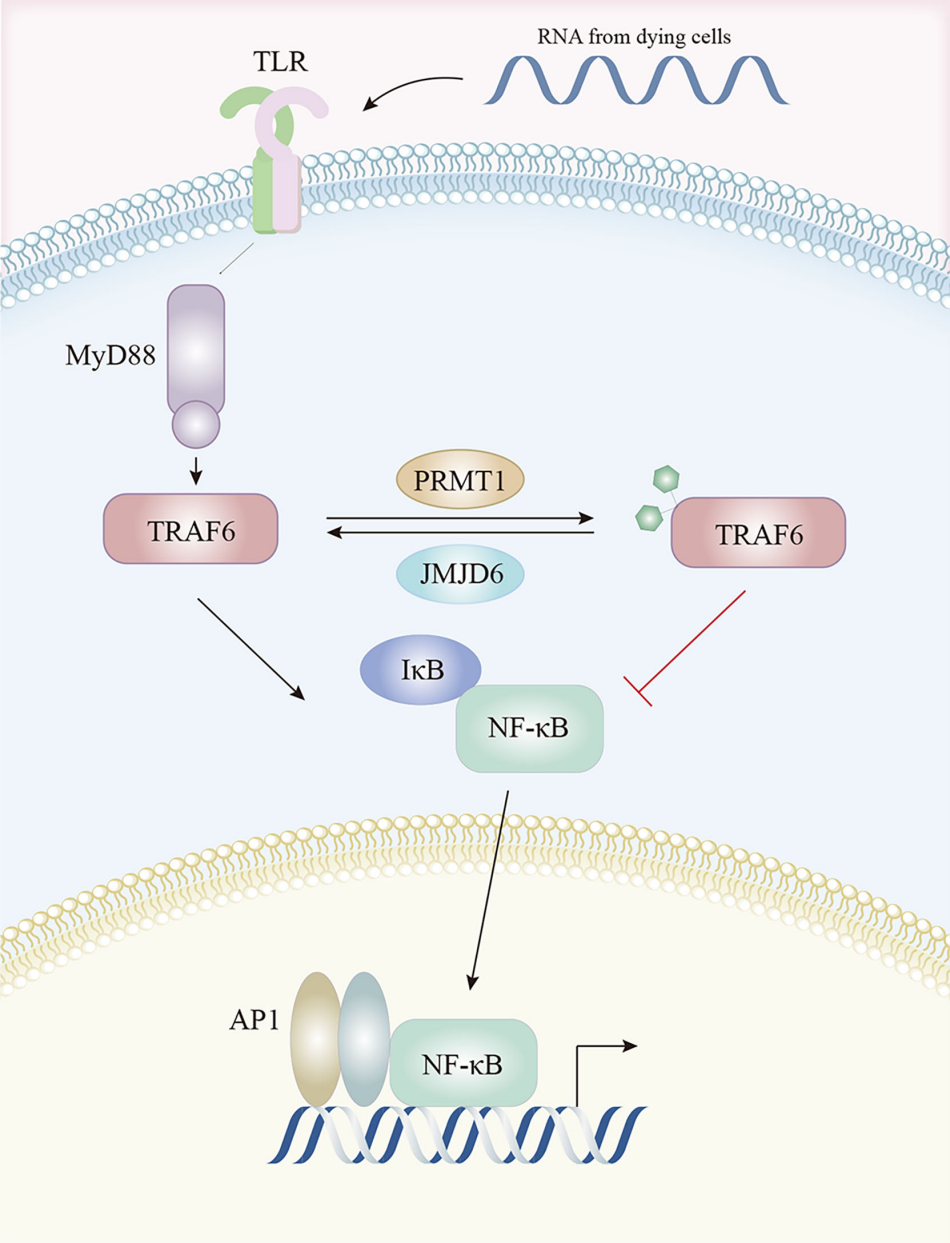
The TLR pathway is regulated by arginine methylation. After activation by TLR ligands, TRAF6 is methylated by PRMT1 and becomes inactive, resulting in lower downstream NF-κB activity. This is reversed by JMJD6 which demethylates TRAF6. Abbreviations: TLR, Toll-like receptors; TRAF6, tumor necrosis factor receptor associated factor 6; NF-κB, nuclear factor kappa-B; JMJD6, Jumonji Domain Containing 6.

Pathogen associated molecular pattern recognition by TLRs contribute to the transcriptional up-regulation of distinct genes. Tumor necrosis factor receptor (TNFR)-associated factor 6 (TRAF6) was identified as key for myeloid differentiation primary response 88 (MyD88)-dependent signaling pathway with immunoregulatory functions, which was a downstream of TLRs (143). PRMT1 methylated and deactivated TRAF6 to suppress the TRAF6-dependent TLR pathway. This suppression was reversed by jumonji domain containing 6 (JMJD6), with TRAF6 demethylated by JMJD6. The PRMT1/JMJD6 ratio determined activation of the MyD88-dependent TLR pathway. The arginine methylation of TRAF6 was associated with lower TRAF6 and downstream NF-κB activity, while exposure to the TLR ligand transiently reduced PRMT1, leading to TRAF6 demethylation by JMJD6 and NF-κB activation, then, once the signal was terminated, PRMT1 re-blocked the TRAF6-dependent pathway (144) (**Figure 3**).

#### 3.2.2 PRMTs and the RIG-I Pathway

**Figure 4:**
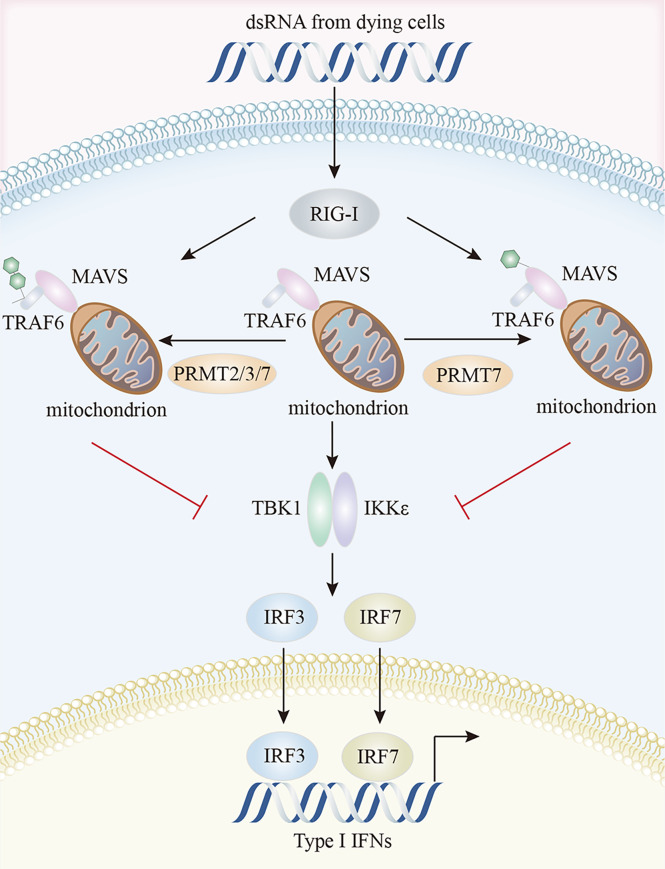
Characteristics of the PRMTs and their interactions in RIG-I pathway. PRMT7 mono-methylates MAVS and inhibits its interaction with RIG-I, inactivating TBK1/IKKϵ signaling and reducing type I IFN production. Additionally, PRMT2, PRMT3, and PRMT7 suppress the auto-ubiquitination of TRAF6 and compete with MAVS for TRAF6 binding, thereby preventing TBK1/IKKϵ recruitment to MAVS. MAVS, mitochondrial antiviral signaling protein; RIG-I, retinoic acid-inducible gene I; TBK1, TANK-binding kinase 1.

In the RIG-I pathway, an important role for PRMT5 was identified in IFI16/IFI204 methylation; IFI16/IFI204 methylation activated dsRNA-induced RIG-I/TLR3-mediated type I interferon responses, even though IFI16/IFI204 usually served a DNA-sensing protein (19, 145). PRMT7 formed aggregates to mono-methylate mitochondrial antiviral-signaling protein (MAVS) at R52, inhibiting its interaction with RIG-I and TRIM31-triggered MAVS K63-linked polyubiquitination in a catalytically-dependent manner. MAVS activation and downstream virus-induced molecular events (IRF3, IκB, and STAT1 phosphorylation) were inhibited by PRMT7, but rescued by the PRMT7 inhibitor, SGC3027 (146). MAVS recruited both TRAF3 and TRAF6 to form the MAVS/TRAF3/TRAF6 complex which interacted with TBK1 and IKK to activate IRF3 and type I IFN production (147). In early virus infection stages, PRMT7 was auto-methylated at R32, disabling its enzymatic activity and aggregation. SMURF1 was then recruited to PRMT7 in a MAVS-dependent manner to catalyze the K48-linked polyubiquitination of PRMT7 to ensure its timely proteasomal degradation and subsequent RIG-I-MAVS activation, while SMURF1 degraded MAVS, TRAF3, TRAF6, or USP25 in later viral infection stages to avoid excessive RLR signaling activation (146). In zebrafish, PRMT2, PRMT3, and PRMT7 attenuated antiviral responses by suppressing RIG-I-MAVS signaling pathway. The Lys63-linked auto-ubiquitination of TRAF6 was prevented by PRMT2-mediated asymmetric dimethylation of R100 at TRAF6, thereby inactivating Nemo-dependent TBK1/IKKϵ signaling (148). PRMT2 also competed with MAVS for TRAF6 binding and prevented TBK1/IKKϵ recruitment to MAVS (148). Through the two aforementioned mechanisms, PRMT2 adversely controlled IRF3/IRF7 phosphorylation and the expression of downstream type I IFN genes (148). These authors also suggested that both PRMT3 and PRMT7 affected IRF3 phosphorylation and suppressed IFN production by interacting with RIG-I (149, 150) (**Figure 4**).

#### 3.2.3 PRMT and Cyclic GMP-AMP Synthase(cGAS)- Stimulator of Interferon Genes (STING) Pathways

**Figure 5:**
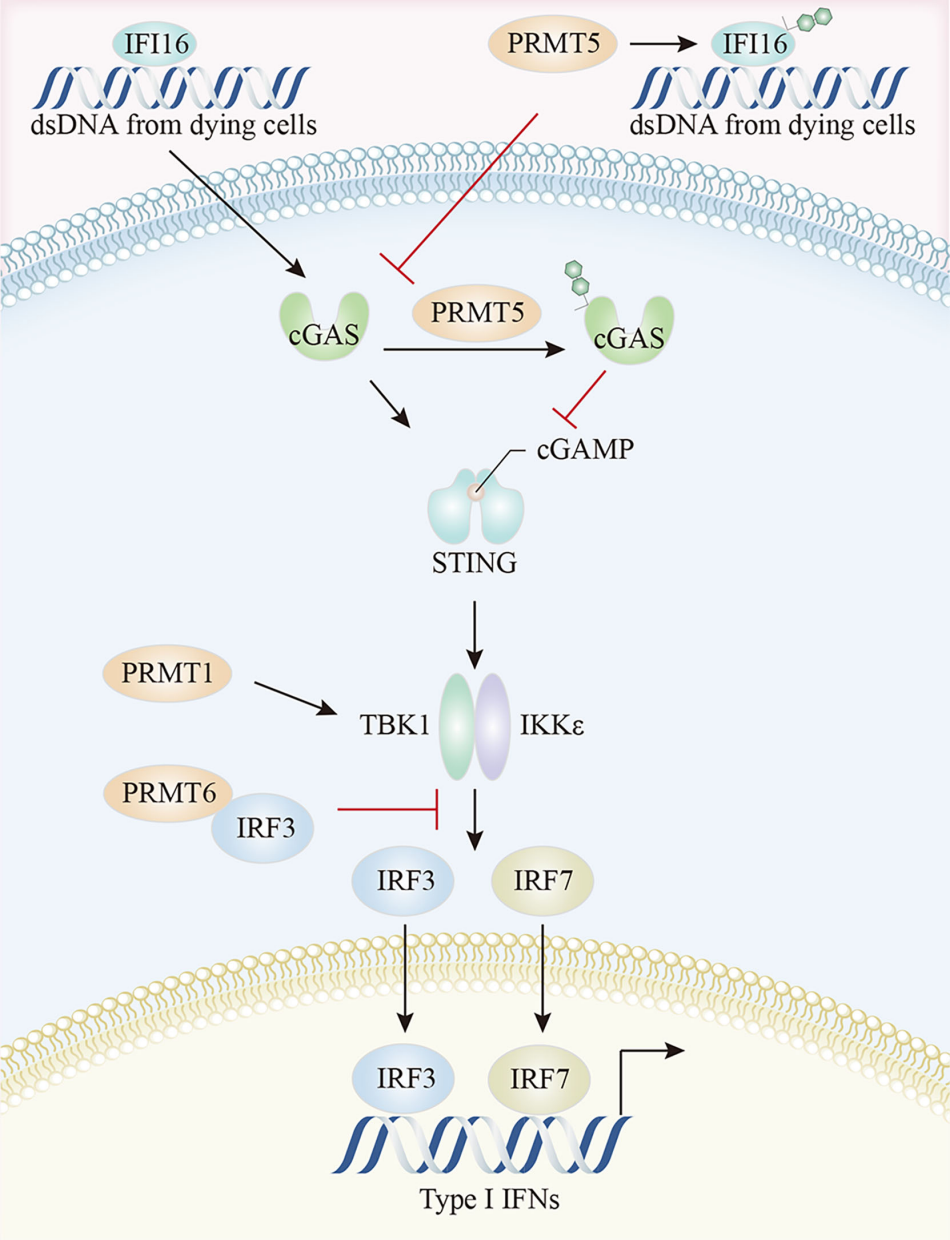
The crosstalk between arginine methylation and cGAS-STING pathway. PRMT5 methylates IFI16, attenuates IFI16 binding to dsDNA, and restrains dsDNA-stimulated activation of cGAS/STING signaling. Moreover, PRMT5 directly interacts with cGAS and impairs cGAS-DNA binding. The arginine methylation of TBK1, catalyzed by PRMT1, enhances TBK1 kinase activity, and PRMT6 inhibits TBK1-IRF3 complex assembly by binding to IRF3, thereby limiting type I IFN production by the TBK1-IRF3 pathway. cGAS, cyclic GMP-AMP synthase; STING, stimulator of interferon genes; TBK1, TANK-binding kinase 1; IRF3, Interferon regulatory Factor 3.

The cGAS-STING pathway is the most compelling activation pathway in tumor innate immunity (151). In melanoma tumor cells, CARM1 ablation induced dsDNA breaks and cGAS-STING activation, together with the increased expression of several ISGs, including *Irf7*, *Ifit1*, *Oasl1*, and *Tap1*, and the enhancement of tumor cell susceptibility to cytotoxic T cells (79). MED12 and TDRD3 are CARM1 effector molecules, which promoted ISG expression, possibly because CARM1 catalyzed MED12 methylation at R1899 which in turn interacted with TDRD3 to facilitate its recruitment. TDRD3 is normally tightly bound to the topoisomerase TOP3B, with the TDRD3-TOP3B complex recruited to the promoter *via* H3R17me2a marks catalyzed by CARM1, to ultimately promote gene expression (79, 135, 152). A study on IFI16/IFI204 methylation in melanoma reported that PRMT5 methylated R12 in the PYRIN (protein-protein interaction) domain of IFI204 *via* a PRMT5-SHARPIN interaction, which attenuated IFI204 binding with dsDNA, restrained dsDNA-stimulated activation of cGAS/STING signaling, and limited subsequent IFN-β and chemokine production by the TBK1-IRF3 pathway (19). It was reported that the PRMT5-MEP50 complex directly interacted with cGAS and catalyzed the R124 dimethylation of cGAS (153). The arginine methylation of cGAS impaired cGAS-DNA binding, attenuated cGAS activation, and inhibited cGAS-STING pathway-mediated type I IFN production, and this enzyme activity-dependent process was rescued by the PRMT5-specific inhibitor, EPZ015666 or PRMT5 specific small interfering RNAs (153) (**Figure 5**). Beyond its well-established role as a general cytosolic DNA sensor, nuclear cGAS has a noncanonical role in response to RNAs *via* PRMT5 recruitment. Specifically, nuclear-localized cGAS facilitated PRMT5 nuclear translocation and its subsequent recruitment to *Ifnb* and *Ifna4* enhancers in a cGAS-dependent manner. PRMT5 then catalyzed the symmetric dimethylation of H3R2me2s to facilitate IRF3 access, thereby enhancing type I IFN production (154).

PRMTs may also regulate downstream TBK1-IRF3 signaling *via* direct interactions. PRMT1 was implicated in TBK1 and IRF3 phosphorylation, IRF3 dimerization, and nuclear translocation. PRMT1 catalyzed TBK1 arginine methylation at R54, R134, and R228 positions, thereby promoting its oligomerization and trans-autophosphorylation. The arginine methylation of TBK1 enhanced its kinase activity, resulting in subsequent type I IFN production, an effect independent of the K63-linked ubiquitination of TBK1 (155). Moreover, PRMT6 regulated IFN-I production by inhibiting TBK1-IRF3 complex assembly rather than TBK1 activity. The N-terminal domain of PRMT6 bound to IRF3, blocking TBK1 and IRF3 interactions, thereby allowing PRMT6 to bind and isolate IRF3 in a manner independent of its methyltransferase activity (154). PRMT6 deficient cells showed enhanced TBK1-IRF3 interactions and subsequent IRF3 activation and type-I IFN production (156).

Additionally, reduced total sDMA levels selectively prevented type I and III IFN production by the context-dependent control of TCR-or PRR-stimulation-dependent transcription of IFNB1 and IFNL1, which was required for ISGF3 complex activation *via* the TBK1-mediated phosphorylation of the AP-1 transcription factors, c-Jun and ATF2 (157). PRMT1 mitigated IFN function by interacting with the IC domain of the IFNα/β receptor IFNAR1 chain (158).

### 3.3 PRMTs and Intrinsic Tumor Resistance Mechanisms

**Figure 6:**
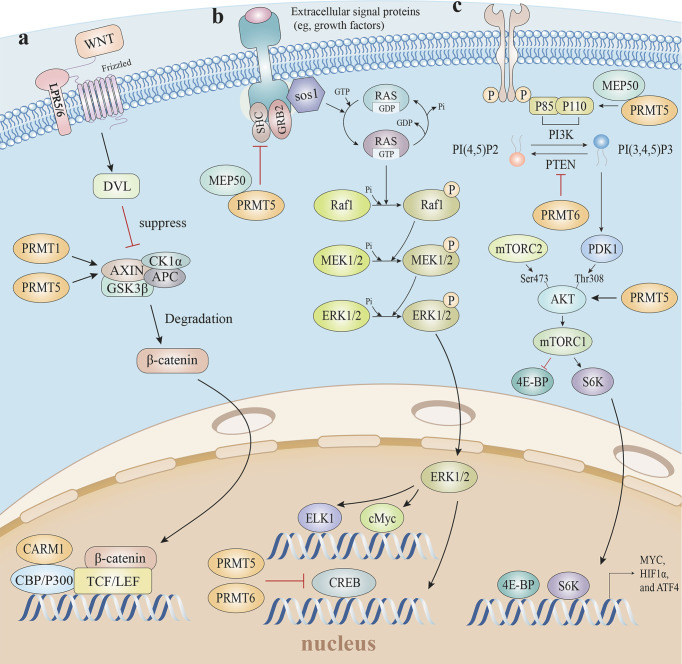
Arginine methylation and related oncogenic pathways. **(A)** PRMT5 increases H3R8 methylation in the promoter region of WNT/β-catenin antagonist genes *Axin1* and *Axin2*, epigenetically suppresses *Axin* expression to promote WNT/β-catenin signaling, and targets gene expression. The PRMT1-mediated methylation of *Axin* decreases ubiquitination and enhances *Axin* stability, which degrades cytoplasmic β-catenin levels. Furthermore, CARM1 and p300 are recruited by β-catenin as coactivators, and H3R17 dimethylation is induced to modulate endogenous WNT target gene expression. **(B)** The mono-methylation of EGFR by the PRMT5-MEP50 complex positively modulates its trans-autophosphorylation at Tyr 1173, which enhances EGFR-SHP1 binding, suppresses SOS phosphorylation, and is followed by EGFR-mediated ERK activation. Additionally, PRMT5 and PRMT6 both methylate CRAF to reduce its stability and catalytic activity, thereby diminishing the amplitude of the ERK1/2 output in the RAS signaling pathway. **(C)** The PRMT5-MEP50 complex methylates PI3K to activate the PI3K/AKT pathway, with PTEN asymmetrically dimethylated by PRMT6 which decreases PTEN phosphatase activity and impedes its ability to inhibit the PI3K-AKT cascade. PRMT5-mediated AKT1 methylation enhances AKT mRNA translation, and PRMT5 is also required for AKT phosphorylation at Thr308 and Ser473, suggesting PRMT5 is an important upstream regulator of Akt, and induces the PI3K/AKT pathway. EGFR, epidermal growth factor receptor; SHP1, SH2-containing protein tyrosine phos-phatase-1; SOS, son of sevenless; ERK, extracellular regulated protein kinases; RAS, rat sarcoma; PI3K, phosphoinositide 3-kinase; AKT, protein kinase B; PTEN, phosphatase and tensin homolog.

Increasing clinical evidence has identified the immunotherapy resistance associated with the activation of particular oncogenic pathways (159). Oncogenes orchestrate immune microenvironments by altering immune cell infiltration and the secretome of cancer cells, while several signaling pathways are involved in ICI resistance (6, 159). Given space limitations, we focus only on WNT/β-catenin, mitogen-activated protein kinases (MAPK), and phosphatase and tensin homolog (PTEN) pathways (**Figure 6**).

#### 3.3.1 PRMTs Regulate the Wnt/β-Catenin Pathway

Blocking Wnt/β-catenin signaling elevated T cell-mediated cytotoxicity levels and boosted T cell infiltration into tumors, leading to complete regression when combined with immunotherapy in the majority of mice in a mouse model study (160). Consistent with non-T-cell-inflamed tumor studies, Wnt/β-catenin signaling drove immune exclusion and potentially served as a molecular target for expanding immunotherapy efficacy (161).

Given the conserved methyltransferase domain is responsible for β-catenin binding, it is possible that β-catenin interacts with many PRMTs (162). For instance, PRMT1 overexpression promoted active β-catenin levels in esophageal carcinoma cells (163). Furthermore, CARM1 and p300, as coactivators, were recruited by β-catenin *via* direct interactions, after which H3R17 dimethylation was induced to modulate endogenous Wnt target gene expression (162). A clinical retrospective and prospective analysis showed that the PRMT5/Wnt4 axis controlled Wnt/β-catenin signaling in laryngeal carcinoma cells by regulating nuclear β-catenin accumulation (164). In chronic myelogenous leukemia cells, PRMT5 activated Wnt/β-catenin signaling by increasing β-catenin and disheveled homolog 3 (DVL3) protein levels, which is an upstream positive β-catenin regulator. PRMT5 was recruited to the *Dvl3* promoter and mediated H3R2me2s to activate *Dvl3* transcription (165). PRMT5 also activated Wnt/β-catenin signaling by the direct epigenetic silencing of the pathway antagonists, AXIN2, WIF1, DKK1, and DKK3. The methylation markers H3R8me2a and H4R3me2a in *Axin2*, *Wif1*, *Dkk1*, and *Dkk3* promoters, and subsequent Wnt/β-catenin signaling restrictions, were decreased in response to PRMT5 inhibition (166). Whereas, PRMT1-mediated methylation of Axin R378 decreased ubiquitination and enhanced Axin stability, which degraded cytoplasmic β-catenin (167). Thus, mounting data suggests arginine methylation exerts substantial and sophisticated roles regulating Wnt/β-catenin signaling pathways.

#### 3.3.2 PRMTs Regulate the MAPK Pathway

Several clinical studies reported that MAP/ERK kinase (MEK) and v-raf murine sarcoma viral oncogene homolog B1 (BRAF) inhibitors, in combination with anti-PD1 therapy, generated long-lasting tumor control due to relative increases in IL-6 and IL-10 expression, and tumor susceptibility to T cell cytotoxic effects (168–170).

MAPK pathway activation was increased in PRMT5 knockout tumor cells. PRMT5 reduced the duration and amplitude of epidermal growth factor (EGF)-mediated ERK activity, and decreased p‐Raf and p‐ERK phosphorylation levels (171, 172). The mono-methylation of epidermal growth factor receptor (EGFR) R1175 by the PRMT5-MEP50 complex in breast cancer favorably controlled its trans-autophosphorylation at Tyr 1173, resulting in endogenous SHP1 recruitment to attenuate son of sevenless (SOS) phosphorylation and ERK activation (173). Consistently, PRMT5 methylated CRAF at R563 which reduced CRAF stability and catalytic activity, thereby diminishing the amplitude of the ERK1/2 output in rat sarcoma (RAS) signaling (174). However, conflicting studies reported the role of PRMT5 in MAPK signaling, which was initiated by RAS-RAF-MEK-ERK stepwise phosphorylation. PRMT5 promoted fibroblast growth factor receptor 3 (FGFR3) expression, which in turn initiated ERK1/2 and PI3K signaling (175). PRMT5 catalyzed H4R3me2s in promoter regions to repress microRNA (miR)-99 transcription, and directly catalyzed the FGFR3 promoter which positively regulated FGFR3-mediated ERK1/2 and AKT activation (176, 177). Except for PRMT5, CRAF was also methylated at R100 by PRMT6, which altered CRAF-RAS binding potential and downstream MEK/ERK signaling activation (178).

#### 3.3.3 PRMTs Regulate the PTEN-PI3K/AKT Pathway

PTEN deletion in melanoma promotes immune resistance, while PI3K-AKT-mTOR inhibitors enhance immunotherapy efficacy by modulating the TME, the mechanisms of which are not clearly understood, but are multifactorial in nature (179, 180). PRMT5 knockdown down-regulated PI3K/AKT/mTOR signaling in an influx of cancer cells, including bladder cancer, lymphoma, and Non-small-cell lung carcer (NSCLC) (181–183). Although links between PRMT5 and PI3K-AKT-mTOR signaling are ubiquitous in numerous cell types, it is unclear how PRMTs affect this pathway; do PRMTs regulate upstream proteins PTEN hypo-phosphorylation, or do PRMTs interact with PI3K/AKT/mTOR directly?

Several studies reported that PRMT5 and PTEN were linked; PRMT5 reduced PTEN mRNA and protein levels in glioblastoma neurospheres (GBMNS), which significantly increased AKT signaling (184). In gastric cancer, PRMT5 directly interacted with c-Myc to transcriptionally repress the expression of c-Myc target genes, including PTEN (138). The PI3K subunit, p55, directly interacted with MEP50 and was methylated by PRMT5 to activate PI3K/AKT signaling (185, 186). In terms of AKT, first, PRMT5 directly methylated AKT1 to promote its activation (187). Second, PRMT5-mediated methylation enhanced AKT mRNA translation, thereby facilitating AKT *de novo* synthesis, which was coordinated by the CITED2-NCL axis (188). Third, PRMT5 elevated AKT phosphorylation *via* the direct transcriptional repression of AXIN2 and WIF1 (166). Fourth, PRMT5 directly co-localized and interacted with AKT, albeit not with PTEN and mTOR; Akt phosphorylation at Thr308 and Ser473 and the downstream target GSK3 at Ser9 was markedly decreased without altering PTEN and mTOR phosphorylation at Ser2442 in PRMT5-deficient lung adenocarcinoma cells (183). Moreover, not only did PRMT5 up-regulate PI3K/AKT signaling, but PI3K/AKT in turn induced PRMT5 expression through the AKT-GSK3β-MYC axis to form a positive-feedback loop (182).

The PI3K-AKT-mTOR pathway was likewise inhibited by other PRMTs. Asymmetrical dimethylation of PTEN R159 by PRMT6 decreased PTEN phosphatase activity and impeded the PI3K-AKT cascade (189). Also, PRMT2 inhibited estrogen receptor-α (ER-α) in breast cancer cells, resulting in the downstream suppression of PI3K/AKT and MAPK/ERK (190).

## 4 PRMTs and Immune Checkpoint Therapy

Of the numerous immune checkpoints, the programmed death ligand-1/programmed death-1 (PD-L1/PD-1) signaling pathway is highly significant as it inhibits TCR-mediated T cell activation to regulate immune responses (191). Antigen-stimulated T cells express PD-1 which is a co-inhibitory receptor that interacts primarily with PD-L1/CD274. This promotes T lymphocyte apoptosis and lymphocyte death primarily by dephosphorylating TCR activation through the tyrosine phosphatase SHP2, thereby inhibiting downstream PI3K/AKT signaling and hindering cytokine secretion by T lymphocytes (191, 192). Moreover, sustained PD-1 signaling was shown to induce metabolic dysregulation that drove CD8^+^ T cell exhaustion (193).

PT1001B (a novel selective inhibitor of type I PRMTs) down-regulated PD-1^+^ leukocytes and reduced PD-L1 expression in a pancreatic cancer mouse model, which significantly improved the inhibition of tumor cell proliferation and apoptosis induction when combined with anti-PD-L1 (194). PRMT1 knockdown in tumor cells and macrophages in a diethylnitrosamine (DEN)-induced hepatocellular carcinoma (HCC) mouse model generated significant decreases in PD-L1 and PD-L2, resulting in reduced therapeutic efficacy of PD-1 antibody treatment (195). Moreover, the PRMT1 gene polymorphism rs975484 may serve as a predictive marker for response to PD-1/PD-L1 treatment (195). In mice implanted with MC38 murine colon adenocarcinoma cells, combining MS023 (a splicing modulator that inhibits type I PRMT enzymes) with PD-1 antibodies provided better therapeutic value (196). The combination of CARM1 inhibitors with CTLA4 or a PD-1 monoclonal antibody increased ICB efficacy in a melanoma mouse model as a result of the dual actions of CARM1 on T and tumor cells (79). As PRMT5 in tumor cells inhibited PD-L1 expression, GSK3326595 (PRMT5 inhibitor) and anti-PD-1 combination therapy was more effective than either treatment alone in murine xenograft liver tumors, a MYC-driven spontaneous HCC model, and murine melanoma models (19, 50). In B16 melanoma cells transfected with PRMT7 small interfering RNA or treated with the PRMT7 small molecular inhibitor, SGC30274, PD-L1 mRNA and protein levels were reduced, and ICI therapy potentiated. This observation could be attributed to increased H4R3me2s levels at the PD-L1 promoter modulated by PRMT7, but also improved IFN-induced PD-L1 expression, as PRMT7 also acted as an IRF-1 co-activator (48). Moreover, a “viral mimicry” response occurred after the up-regulation of endogenous retroviral element transcription, dsRNA expression, and stress granule formation due to diminished DNMT expression in the absence of PRMT7, thereby causing IFN activation and immune cell infiltration in B16F10 cells (48).

Numerous cytokines interact with PRMTs to maintain PD-L1 expression, the most efficient of which is IFN-γ. IFN-γ uses multiple pathways to induce PD-L1 expression in different tumor types, including JAK2/STAT1/IFR-1 pathways in gastric cancer, JAK/STAT3 and PI3K-AKT pathways in lung cancer, and MyD88-, TRAF6-, and MEK-dependent pathways in myeloma (197–199). PRMT activity inhibition blunted the IFN-γ secretion (86, 200–202). PRMT1 also methylated the NFAT cofactor protein NIP45 to augment IFN-γ production (90). In the TME of a PRMT5 knockdown transplanted tumor model, PD-1 and TIM-3 expression and function were both inhibited in CD8^+^ T cells. PRMT5 inhibition suppressed STAT1 phosphorylation both *in vivo* and *in vitro*, and was accompanied by decreased IFN-γ production by T cells, and ISG transcription (200). One reason for this was that PRMT5 induced H3R2me2s marker enrichment in the STAT1 promoter region, between -1267 bp and -1094 bp, to enhance PD-L1 expression *via* the IFNγ/JAK/STAT1 axis. The other reason was that PRMT5 bound to the PD-L1 promoter region between -792 bp and -671 bp, and directly activated its transcription *via* an unknown transcription factor (203).

## 5 Conclusions and Perspectives

Accumulating evidence now links PRMTs to anti-cancer immune alterations, where they exhibit multiple pleiotropic effects that facilitate other modifications beyond their immediate targets. Specifically, key PRMT influences on the cancer-immunity cycle and cancer immunotherapy have been demonstrated. PRMT5 restricted antigen processing and presentation in combination with inhibiting the cell surface expression of MHC I by modulating NLRC5 and IRF expression (19, 39, 45). Due to the conservation of catalytic sites, PRMT1, PRMT5, and CARM1 all promoted CXCL10 and CXCL11 transcriptional expression, while PRMT chemokine regulation was contextually relevant as PRMTs recruited different transcription factors at different stages during biological responses (56–60). PRMT-mediated histone post-translational modifications have irreplaceable roles in initiating and activating T and B cells, TAM differentiation, the inhibitory effects of FOXP3+ Treg cells, and the induction of PD-L1 checkpoints. Additionally, PRMT-mediated chromatin remodeling contributed to the cytotoxic and depleted phenotypes of tumor-infiltrating CD8^+^ T cells. Therefore, PRMT inhibitors may be effective not just for ICB therapy, but also alternative immunotherapies where T cells function as key effector cells, such as neoantigen-based cancer vaccines and chimeric antigen receptor T-cell therapies. Also, PRMT inhibition altered intrinsic tumor cell pathways, such as activating WNT-β catenin signaling to blunt T cell priming and recruitment, or suppressing PTEN to impair T cell-mediated killing, to indirectly regulate the immune microenvironment.

As methylation is a targetable modification, several studies have investigated the therapeutic potential of PRMTs in preclinical models, and their underlying associations with tumorigenesis in animal models. These studies established a rationale for using inhibitors against PRMT5 and type I PRMTs in clinical trials. Thus far, such inhibitors have been tested in patients with hematological or solid tumors (204). GSK3326595 is a selective PRMT5 inhibitor and was used in the METEOR-1 phase I study to investigate the safety, pharmacokinetics, pharmacodynamics, and efficacy of GSK3326595 in adults with solid tumors and non-Hodgkin lymphoma. Critically, patients showed promising responses to therapy, and adverse events were prevalent but manageable (205). Furthermore, forthcoming research programs from this trial will include GSK3326595 and pembrolizumab combination therapy to investigate the efficacy of PRMT5 inhibitor and immunotherapy combination (205). In addition, another type I PRMT inhibitor, GSK3368715 (EPZ019997), induced anti-tumor effects over a broad range of hematological and solid tumor types, especially S-methyl-5’-thioadenosine phosphorylase gene (MTAP) -deficient tumors (NCT03666988) (204). Despite these advances, further investigations are required to address the many limitations, including potential toxicity over time, contrast targets or responses in specific cancer types, and compensatory mechanisms in PRMTs to improve all therapeutic modalities. Currently, only four PRMT inhibitor based clinical cancer trials have been reported (https://www.clinicaltrials.gov/): PRMT1 inhibitor GSK3368715, and PRMT5 inhibitors GSK3326595, JNJ-64619178, and PF-06939999. While some clinical trials reported encouraging results, considerable uncertainty remains in terms of inhibitor safety, tolerability, pharmacokinetic profiles, and the combined therapeutic benefit of inhibitors and immunotherapy for cancer patients. Therefore, comprehensive pharmacokinetic and pharmacodynamic evaluations are required to maximize therapeutic efficacy while minimizing toxicity.

Overall, our understanding of PRMT functions and mechanisms in tumor immunity is in its infancy, however, several intriguing and critical questions require answers, 1) what are the epigenetic modification mechanisms associated with activated phenotypes in adaptive immune cells, 2) what is the immunological relevance of crosstalk between PRMTs, 3) what are their regulators, co-activators, targets, and molecular interactions, and 4) how do we integrate PRMT inhibitors with immunotherapies to achieve maximal and permanent therapeutic effects for cancer patients. Technological developments such as CRISPR-Cas9 based screens to identify immunological-related genes, and transcriptome single cell sequencing of tumor-infiltrating immune cells may shed light on how PRMTs regulate TME phenotypes and function, which typically has been limited to small molecule inhibitors or transgenic mouse models rather than the genome-scale screening of primary immune cells. Similarly, next-generation sequencing technologies and small molecule inhibitor therapies, with improved specificity and affinity, will undoubtedly refine our understanding of arginine methylation mechanisms in unravelling antitumor immunity in different tumor types at different clinical stages.

PRMT inhibitors may function as a double-edged sword; they may selectively enhance or severely interfere with key aspects of antitumor immune responses, with unknown impacts on therapeutic success. Therefore, in developing cancer-specific therapeutic strategies for reprogramming immune responses against PRMT targets, careful rational drug combinations and regimens are required in combination with innovative multi-target strategies that circumvent adaptive resistance mechanisms. This way, we can improve the prognosis of multiple cancers, especially those that are immunotherapy negative.

## Author Contributions

WD, JZ, SL, FT, CX, and ZW designed and wrote the article. FH, QL, ZY, JG, and YG critically revised the article. All authors contributed to the article and approved the submitted version.

## Funding

This study was supported by the National Natural Science Foundation of China (grant nos. 81773236, 81800429 and 81972852), the Key Research & Development Project of Hubei Province (grant no. 2020BCA069), the Nature Science Foundation of Hubei Province (grant no. 2020CFB612), the Health Commission of Hubei Province Medical Leading Talent Project, Young and Middle−Aged Medical Backbone Talents of Wuhan (grant no. WHQG201902), the Application Foundation Frontier Project of Wuhan (grant no. 2020020601012221), the Zhongnan Hospital of Wuhan University Talented Doctor Program (grant nos. ZNYB2021008), the Zhongnan Hospital of Wuhan University Medical Science and Technology Innovation Platform Program (grant nos. PTXM2022025), the Zhongnan Hospital of Wuhan University Science, Technology and Innovation Seed Fund (grant nos. znpy2019001 and znpy2019048) and the Translational Medicine and Interdisciplinary Research Joint Fund of Zhongnan Hospital of Wuhan University (grant nos. ZNJC201922 and ZNJC202007).

## Conflict of Interest

The authors declare that the research was conducted in the absence of any commercial or financial relationships that could be construed as a potential conflict of interest.

The reviewer JZ declared a shared affiliation with the authors to the handling editor at time of review.

## Publisher’s Note

All claims expressed in this article are solely those of the authors and do not necessarily represent those of their affiliated organizations, or those of the publisher, the editors and the reviewers. Any product that may be evaluated in this article, or claim that may be made by its manufacturer, is not guaranteed or endorsed by the publisher.
