# Supplementary material for: Protein Arginine Methylation: An Emerging Modification in Cancer Immunity and Immunotherapy
Source: Front Immunol. 2022 Apr 14;13:865964. doi: 10.3389/fimmu.2022.865964 (PMC9046588; doi:10.3389/fimmu.2022.865964)
Supplement: Supplementary file 1 [file Table_1.docx]

**Supplementary Table1**

The major substrates and their function of PRMTs.

| **Enzymes** | **Substrates** | **Biological function** | **Ref** |
| --- | --- | --- | --- |
| PRMT1 | H4R3me2a | Activate transcription via increasing p300 acetylation | (1,2) |
|  | STAT1 | Activate IFN alpha/beta-induced transcription | (3) |
|  | p14^ARF^ | Promote p53-independent apoptosis | (4) |
|  | LCN2 | Facilitate sunitinib-induced upregulation of LCN2-AKT-RB signaling | (5) |
|  | C/EBPα | Promote the expression of cyclin D1 | (6) |
|  | RUNX1 | Activate transcription via shedding corepressors and recruiting coactivators | (7) |
|  | FOXO1 | Block Akt-mediated phosphorylation of FOXO1 | (8) |
|  | Twist1 | Activate E-cadherin repression | (9) |
|  | p65 | Block TNFα-induced activation of NF-κB | (10) |
|  | Gli1 | Activate transcriptional activity of Gli1 | (11) |
|  | hnRNP K | Block activation of c-Src | (12) |
|  | RBM15 | Regulate alternative RNA splicing via reducing RBM15 protein | (13) |
|  | EGFR | Promote receptor dimerization and signaling activation | (14) |
|  | ASK1 | Attenuate paclitaxel-induced apoptosis | (15) |
|  | SMAD7 | Facilitate TGF-β signaling | (16) |
|  | CDK4 | Block the formation of a CDK4-Cyclin-D3 complex | (17) |
|  | RACO-1 | Stabilize RACO-1 and enable RACO-1 dimerization | (18) |
|  | TOP3B | Attenuate the accumulation of R-loops | (19) |
| PRMT2 | H3R8me2a | Activate Wnt/β-catenin signaling | (20,21) |
|  | ER-α | Block the expression of cyclin D1 | (22) |
| PRMT3 | H4R3me2a | Promote the expression of miR-3648 | (23) |
|  | ALDH1A1 | Block the expression of retinoic acid responsive genes | (24) |
|  | LXRα | Induce lipogenesis | (25) |
|  | TOP3B | Attenuate the accumulation of R-loops | (19) |
| CARM1 | H3R17me2a | Activate transcription | (26) |
|  | H3R26me2a | Activate and repress transcription | (27,28) |
|  | H3R42me2a | Activate transcription | (29) |
|  | MED12 | Block p21/WAF1 transcription and activate ER-α mediated gene transcription | (30,31) |
|  | PARP1 | Facilitate replication fidelity | (32) |
|  | GAPDH | Inhibit glycolysis | (33) |
|  | BAF155 | Activate transcription and displace BAF155 by EZH2 | (34,35) |
|  | PKM2 | Facilitate aerobic glycolysis | (36) |
|  | MDH1 | Repress mitochondria respiration and inhibits glutamine metabolism | (37) |
|  | p54(nrb) | Impair binding of p54(nrb) to mRNAs containing IRAlus | (38) |
|  | HSP70 | Regulate RARβ2 gene transcription | (39) |
|  | RUNX1 | Promote the expression of miR-223 | (40) |
|  | p300 | Impair binding of p300 to ACT | (41) |
|  | HuR | Regulate subcellular localization | (42) |
|  | LSD1 | Promote deubiquitination and stabilization of LSD1 | (43) |
|  | Pax7 | Reduce transcription of Myf5 and other Pax7 target genes | (44) |
| PRMT5 | H4R3me2s | Transcription repression | (45) |
|  | H3R2me2s | Transcription activation | (46) |
|  | H3R8me2s | Transcription repression | (47) |
|  | RNAP II | Attenuate the accumulation of R-loops | (48) |
|  | p65 | Promote NF-κB-induced gene expression | (49) |
|  | Sm proteins | Facilitate spliceosomal assembly | (50) |
|  | p53 | Facilitate cell-cycle arrest. | (51) |
|  | FEN1 | Facilitate PCNA interaction and DNA repair | (52) |
|  | BCL6 | Promote GC formation | (53) |
|  | Nucleolin | Transcription activation | (54) |
|  | EGFR | Attenuate EGFR-mediated ERK activation | (55) |
|  | cGAS | Block the DNA binding ability of cGAS | (56) |
|  | ZNF326 | Impair alternative splicing | (57) |
|  | hnRNP A1 | Promote IRES-dependent translation | (58) |
|  | CRAF | Enhance the degradation of activated CRAF | (59) |
|  | 53BP1 | Promote NHEJ repair by stabilizing 53BP1 | (60) |
|  | Rad9 | Resist to DNA damaging stresses. | (61) |
|  | E2F1 | Promote proliferation | (62) |
|  | KLF4 | Inhibit KLF4 ubiquitylation by VHL | (63) |
|  | RUVBL1 | Promote HR | (64) |
| PRMT6 | H3R2me2a | Repress transcription and facilitate chromosome condensation | (65,66) |
|  | H3R42me2a | Activate transcription | (29) |
|  | TOP3B | Attenuate the accumulation of R-loops | (19) |
|  | CRAF | Impair RAS binding affinity and downstream MEK/ERK signaling | (67) |
|  | RCC1 | Promote RCC1 association with chromatin | (68) |
|  | FOXO3 | Regulate autophagy and protein degradation | (69) |
|  | BAG5 | Enhance the degradation of HSC70 | (70) |
|  | IRF3 | Inhibit the interaction between IRF3 and TBK1 | (71) |
| PRMT7 | H4R3me2s | Repress the miR-24-2 gene | (72) |
|  | p38MAPK | Promote the expression of PGC-1α | (73) |
|  | MRPS23 | Accelerate the degradation of MRPS23 | (74) |
|  | YY1 and HDAC3 | Inhibit the expression of E-cadherin | (75) |
| PRMT9 | SAP145 | Promote the U2 snRNP for interaction with SMN. | (76) |

**References**

1. Wang H, Huang ZQ, Xia L, Feng Q, Erdjument-Bromage H, Strahl BD, Briggs SD, Allis CD, Wong J, Tempst P, et al. Methylation of histone H4 at arginine 3 facilitating transcriptional activation by nuclear hormone receptor. *Science* (2001) **293**:853–857. doi: 10.1126/science.1060781

2. Li X, Hu X, Patel B, Zhou Z, Liang S, Ybarra R, Qiu Y, Felsenfeld G, Bungert J, Huang S. H4R3 methylation facilitates beta-globin transcription by regulating histone acetyltransferase binding and H3 acetylation. *Blood* (2010) **115**:2028–2037. doi: 10.1182/blood-2009-07-236059

3. Mowen KA, Tang J, Zhu W, Schurter BT, Shuai K, Herschman HR, David M. Arginine methylation of STAT1 modulates IFNalpha/beta-induced transcription. *Cell* (2001) **104**:731–741. doi: 10.1016/s0092-8674(01)00269-0

4. Repenning A, Happel D, Bouchard C, Meixner M, Verel-Yilmaz Y, Raifer H, Holembowski L, Krause E, Kremmer E, Feederle R, et al. PRMT1 promotes the tumor suppressor function of p14ARF and is indicative for pancreatic cancer prognosis. *EMBO J* (2021) **40**:e106777. doi: 10.15252/embj.2020106777

5. Wang J, Wang C, Xu P, Li X, Lu Y, Jin D, Yin X, Jiang H, Huang J, Xiong H, et al. PRMT1 is a novel molecular therapeutic target for clear cell renal cell carcinoma. *Theranostics* (2021) **11**:5387–5403. doi: 10.7150/thno.42345

6. Liu L-M, Sun W-Z, Fan X-Z, Xu Y-L, Cheng M-B, Zhang Y. Methylation of C/EBPα by PRMT1 Inhibits Its Tumor-Suppressive Function in Breast Cancer. *Cancer Res* (2019) **79**:2865–2877. doi: 10.1158/0008-5472.CAN-18-3211

7. Zhao X, Jankovic V, Gural A, Huang G, Pardanani A, Menendez S, Zhang J, Dunne R, Xiao A, Erdjument-Bromage H, et al. Methylation of RUNX1 by PRMT1 abrogates SIN3A binding and potentiates its transcriptional activity. *Genes Dev* (2008) **22**:640–653. doi: 10.1101/gad.1632608

8. Yamagata K, Daitoku H, Takahashi Y, Namiki K, Hisatake K, Kako K, Mukai H, Kasuya Y, Fukamizu A. Arginine methylation of FOXO transcription factors inhibits their phosphorylation by Akt. *Mol Cell* (2008) **32**:221–231. doi: 10.1016/j.molcel.2008.09.013

9. Avasarala S, Van Scoyk M, Karuppusamy Rathinam MK, Zerayesus S, Zhao X, Zhang W, Pergande MR, Borgia JA, DeGregori J, Port JD, et al. PRMT1 Is a Novel Regulator of Epithelial-Mesenchymal-Transition in Non-small Cell Lung Cancer. *J Biol Chem* (2015) **290**:13479–13489. doi: 10.1074/jbc.M114.636050

10. Reintjes A, Fuchs JE, Kremser L, Lindner HH, Liedl KR, Huber LA, Valovka T. Asymmetric arginine dimethylation of RelA provides a repressive mark to modulate TNFα/NF-κB response. *Proc Natl Acad Sci U S A* (2016) **113**:4326–4331. doi: 10.1073/pnas.1522372113

11. Wang Y, Hsu J-M, Kang Y, Wei Y, Lee P-C, Chang S-J, Hsu Y-H, Hsu JL, Wang H-L, Chang W-C, et al. Oncogenic Functions of Gli1 in Pancreatic Adenocarcinoma Are Supported by Its PRMT1-Mediated Methylation. *Cancer Res* (2016) **76**:7049–7058. doi: 10.1158/0008-5472.CAN-16-0715

12. Ostareck-Lederer A, Ostareck DH, Rucknagel KP, Schierhorn A, Moritz B, Huttelmaier S, Flach N, Handoko L, Wahle E. Asymmetric arginine dimethylation of heterogeneous nuclear ribonucleoprotein K by protein-arginine methyltransferase 1 inhibits its interaction with c-Src. *J Biol Chem* (2006) **281**:11115–11125. doi: 10.1074/jbc.M513053200

13. Zhang L, Tran N-T, Su H, Wang R, Lu Y, Tang H, Aoyagi S, Guo A, Khodadadi-Jamayran A, Zhou D, et al. Cross-talk between PRMT1-mediated methylation and ubiquitylation on RBM15 controls RNA splicing. *Elife* (2015) **4**:e07938. doi: 10.7554/eLife.07938

14. Liao H-W, Hsu J-M, Xia W, Wang H-L, Wang Y-N, Chang W-C, Arold ST, Chou C-K, Tsou P-H, Yamaguchi H, et al. PRMT1-mediated methylation of the EGF receptor regulates signaling and cetuximab response. *J Clin Invest* (2015) **125**:4529–4543. doi: 10.1172/JCI82826

15. Cho J-H, Lee M-K, Yoon KW, Lee J, Cho S-G, Choi E-J. Arginine methylation-dependent regulation of ASK1 signaling by PRMT1. *Cell Death Differ* (2012) **19**:859–870. doi: 10.1038/cdd.2011.168

16. Katsuno Y, Qin J, Oses-Prieto J, Wang H, Jackson-Weaver O, Zhang T, Lamouille S, Wu J, Burlingame A, Xu J, et al. Arginine methylation of SMAD7 by PRMT1 in TGF-β-induced epithelial-mesenchymal transition and epithelial stem-cell generation. *J Biol Chem* (2018) **293**:13059–13072. doi: 10.1074/jbc.RA118.002027

17. Dolezal E, Infantino S, Drepper F, Börsig T, Singh A, Wossning T, Fiala GJ, Minguet S, Warscheid B, Tarlinton DM, et al. The BTG2-PRMT1 module limits pre-B cell expansion by regulating the CDK4-Cyclin-D3 complex. *Nat Immunol* (2017) **18**:911–920. doi: 10.1038/ni.3774

18. Davies CC, Chakraborty A, Diefenbacher ME, Skehel M, Behrens A. Arginine methylation of the c-Jun coactivator RACO-1 is required for c-Jun/AP-1 activation. *EMBO J* (2013) **32**:1556–1567. doi: 10.1038/emboj.2013.98

19. Huang L, Wang Z, Narayanan N, Yang Y. Arginine methylation of the C-terminus RGG motif promotes TOP3B topoisomerase activity and stress granule localization. *Nucleic Acids Res* (2018) **46**:3061–3074. doi: 10.1093/nar/gky103

20. Blythe SA, Cha S-W, Tadjuidje E, Heasman J, Klein PS. beta-Catenin primes organizer gene expression by recruiting a histone H3 arginine 8 methyltransferase, Prmt2. *Dev Cell* (2010) **19**:220–231. doi: 10.1016/j.devcel.2010.07.007

21. Su X, Zhu G, Ding X, Lee SY, Dou Y, Zhu B, Wu W, Li H. Molecular basis underlying histone H3 lysine-arginine methylation pattern readout by Spin/Ssty repeats of Spindlin1. *Genes Dev* (2014) **28**:622–636. doi: 10.1101/gad.233239.113

22. Zhong J, Cao R-X, Liu J-H, Liu Y-B, Wang J, Liu L-P, Chen Y-J, Yang J, Zhang Q-H, Wu Y, et al. Nuclear loss of protein arginine N-methyltransferase 2 in breast carcinoma is associated with tumor grade and overexpression of cyclin D1 protein. *Oncogene* (2014) **33**:5546–5558. doi: 10.1038/onc.2013.500

23. Min Z, Xiaomeng L, Zheng L, Yangge D, Xuejiao L, Longwei L, Xiao Z, Yunsong L, Ping Z, Yongsheng Z. Asymmetrical methyltransferase PRMT3 regulates human mesenchymal stem cell osteogenesis via miR-3648. *Cell Death Dis* (2019) **10**:581. doi: 10.1038/s41419-019-1815-7

24. Verma M, Khan MIK, Kadumuri RV, Chakrapani B, Awasthi S, Mahesh A, Govindaraju G, Chavali PL, Rajavelu A, Chavali S, et al. PRMT3 interacts with ALDH1A1 and regulates gene-expression by inhibiting retinoic acid signaling. *Commun Biol* (2021) **4**:109. doi: 10.1038/s42003-020-01644-3

25. Kim D, Park M, Lim S, Park J, Yoon K, Han H, Gustafsson J-åke, Lim J, Park S. PRMT3 regulates hepatic lipogenesis through direct interaction with LXRα. *Diabetes* (2015) **64**:60–71. doi: 10.2337/db13-1394

26. Daujat S, Bauer U-M, Shah V, Turner B, Berger S, Kouzarides T. Crosstalk between CARM1 methylation and CBP acetylation on histone H3. *Curr Biol* (2002) **12**:2090–2097. doi: 10.1016/s0960-9822(02)01387-8

27. Goolam M, Scialdone A, Graham SJL, Macaulay IC, Jedrusik A, Hupalowska A, Voet T, Marioni JC, Zernicka-Goetz M. Heterogeneity in Oct4 and Sox2 Targets Biases Cell Fate in 4-Cell Mouse Embryos. *Cell* (2016) **165**:61–74. doi: 10.1016/j.cell.2016.01.047

28. Zhang Z, Nikolai BC, Gates LA, Jung SY, Siwak EB, He B, Rice AP, O’Malley BW, Feng Q. Crosstalk between histone modifications indicates that inhibition of arginine methyltransferase CARM1 activity reverses HIV latency. *Nucleic Acids Res* (2017) **45**:9348–9360. doi: 10.1093/nar/gkx550

29. Casadio F, Lu X, Pollock SB, LeRoy G, Garcia BA, Muir TW, Roeder RG, Allis CD. H3R42me2a is a histone modification with positive transcriptional effects. *Proc Natl Acad Sci U S A* (2013) **110**:14894–14899. doi: 10.1073/pnas.1312925110

30. Wang L, Zeng H, Wang Q, Zhao Z, Boyer TG, Bian X, Xu W. MED12 methylation by CARM1 sensitizes human breast cancer cells to chemotherapy drugs. *Sci Adv* (2015) **1**:e1500463. doi: 10.1126/sciadv.1500463

31. Peng B-L, Li W-J, Ding J-C, He Y-H, Ran T, Xie B-L, Wang Z-R, Shen H-F, Xiao R-Q, Gao W-W, et al. A hypermethylation strategy utilized by enhancer-bound CARM1 to promote estrogen receptor α-dependent transcriptional activation and breast carcinogenesis. *Theranostics* (2020) **10**:3451–3473. doi: 10.7150/thno.39241

32. Genois M-M, Gagné J-P, Yasuhara T, Jackson J, Saxena S, Langelier M-F, Ahel I, Bedford MT, Pascal JM, Vindigni A, et al. CARM1 regulates replication fork speed and stress response by stimulating PARP1. *Mol Cell* (2021) **81**:784-800.e8. doi: 10.1016/j.molcel.2020.12.010

33. Zhong X-Y, Yuan X-M, Xu Y-Y, Yin M, Yan W-W, Zou S-W, Wei L-M, Lu H-J, Wang Y-P, Lei Q-Y. CARM1 Methylates GAPDH to Regulate Glucose Metabolism and Is Suppressed in Liver Cancer. *Cell Rep* (2018) **24**:3207–3223. doi: 10.1016/j.celrep.2018.08.066

34. Wang L, Zhao Z, Meyer MB, Saha S, Yu M, Guo A, Wisinski KB, Huang W, Cai W, Pike JW, et al. CARM1 methylates chromatin remodeling factor BAF155 to enhance tumor progression and metastasis. *Cancer Cell* (2014) **25**:21–36. doi: 10.1016/j.ccr.2013.12.007

35. Karakashev S, Zhu H, Wu S, Yokoyama Y, Bitler BG, Park P-H, Lee J-H, Kossenkov AV, Gaonkar KS, Yan H, et al. CARM1-expressing ovarian cancer depends on the histone methyltransferase EZH2 activity. *Nat Commun* (2018) **9**:631. doi: 10.1038/s41467-018-03031-3

36. Liu F, Ma F, Wang Y, Hao L, Zeng H, Jia C, Wang Y, Liu P, Ong IM, Li B, et al. PKM2 methylation by CARM1 activates aerobic glycolysis to promote tumorigenesis. *Nat Cell Biol* (2017) **19**:1358–1370. doi: 10.1038/ncb3630

37. Wang Y-P, Zhou W, Wang J, Huang X, Zuo Y, Wang T-S, Gao X, Xu Y-Y, Zou S-W, Liu Y-B, et al. Arginine Methylation of MDH1 by CARM1 Inhibits Glutamine Metabolism and Suppresses Pancreatic Cancer. *Mol Cell* (2016) **64**:673–687. doi: 10.1016/j.molcel.2016.09.028

38. Hu S-B, Xiang J-F, Li X, Xu Y, Xue W, Huang M, Wong CC, Sagum CA, Bedford MT, Yang L, et al. Protein arginine methyltransferase CARM1 attenuates the paraspeckle-mediated nuclear retention of mRNAs containing IRAlus. *Genes Dev* (2015) **29**:630–645. doi: 10.1101/gad.257048.114

39. Gao W, Xiao R, Peng B, Xu H, Shen H, Huang M, Shi T, Yi J, Zhang W, Wu X, et al. Arginine methylation of HSP70 regulates retinoid acid-mediated RARβ2 gene activation. *Proc Natl Acad Sci U S A* (2015) **112**:E3327-3336. doi: 10.1073/pnas.1509658112

40. Vu LP, Perna F, Wang L, Voza F, Figueroa ME, Tempst P, Erdjument-Bromage H, Gao R, Chen S, Paietta E, et al. PRMT4 blocks myeloid differentiation by assembling a methyl-RUNX1-dependent repressor complex. *Cell Rep* (2013) **5**:1625–1638. doi: 10.1016/j.celrep.2013.11.025

41. Bao J, Rousseaux S, Shen J, Lin K, Lu Y, Bedford MT. The arginine methyltransferase CARM1 represses p300•ACT•CREMτ activity and is required for spermiogenesis. *Nucleic Acids Res* (2018) **46**:4327–4343. doi: 10.1093/nar/gky240

42. Battaglia-Hsu S-F, Ghemrawi R, Coelho D, Dreumont N, Mosca P, Hergalant S, Gauchotte G, Sequeira JM, Ndiongue M, Houlgatte R, et al. Inherited disorders of cobalamin metabolism disrupt nucleocytoplasmic transport of mRNA through impaired methylation/phosphorylation of ELAVL1/HuR. *Nucleic Acids Res* (2018) **46**:7844–7857. doi: 10.1093/nar/gky634

43. Liu J, Feng J, Li L, Lin L, Ji J, Lin C, Liu L, Zhang N, Duan D, Li Z, et al. Arginine methylation-dependent LSD1 stability promotes invasion and metastasis of breast cancer. *EMBO Rep* (2020) **21**:e48597. doi: 10.15252/embr.201948597

44. Chang NC, Sincennes M-C, Chevalier FP, Brun CE, Lacaria M, Segalés J, Muñoz-Cánoves P, Ming H, Rudnicki MA. The Dystrophin Glycoprotein Complex Regulates the Epigenetic Activation of Muscle Stem Cell Commitment. *Cell Stem Cell* (2018) **22**:755-768.e6. doi: 10.1016/j.stem.2018.03.022

45. Zhao Q, Rank G, Tan YT, Li H, Moritz RL, Simpson RJ, Cerruti L, Curtis DJ, Patel DJ, Allis CD, et al. PRMT5-mediated methylation of histone H4R3 recruits DNMT3A, coupling histone and DNA methylation in gene silencing. *Nat Struct Mol Biol* (2009) **16**:304–311. doi: 10.1038/nsmb.1568

46. Chiang K, Zielinska AE, Shaaban AM, Sanchez-Bailon MP, Jarrold J, Clarke TL, Zhang J, Francis A, Jones LJ, Smith S, et al. PRMT5 Is a Critical Regulator of Breast Cancer Stem Cell Function via Histone Methylation and FOXP1 Expression. *Cell Rep* (2017) **21**:3498–3513. doi: 10.1016/j.celrep.2017.11.096

47. Tae S, Karkhanis V, Velasco K, Yaneva M, Erdjument-Bromage H, Tempst P, Sif S. Bromodomain protein 7 interacts with PRMT5 and PRC2, and is involved in transcriptional repression of their target genes. *Nucleic Acids Res* (2011) **39**:5424–5438. doi: 10.1093/nar/gkr170

48. Zhao DY, Gish G, Braunschweig U, Li Y, Ni Z, Schmitges FW, Zhong G, Liu K, Li W, Moffat J, et al. SMN and symmetric arginine dimethylation of RNA polymerase II C-terminal domain control termination. *Nature* (2016) **529**:48–53. doi: 10.1038/nature16469

49. Wei H, Wang B, Miyagi M, She Y, Gopalan B, Huang D-B, Ghosh G, Stark GR, Lu T. PRMT5 dimethylates R30 of the p65 subunit to activate NF-κB. *Proc Natl Acad Sci U S A* (2013) **110**:13516–13521. doi: 10.1073/pnas.1311784110

50. Gonsalvez GB, Tian L, Ospina JK, Boisvert F-M, Lamond AI, Matera AG. Two distinct arginine methyltransferases are required for biogenesis of Sm-class ribonucleoproteins. *J Cell Biol* (2007) **178**:733–740. doi: 10.1083/jcb.200702147

51. Jansson M, Durant ST, Cho E-C, Sheahan S, Edelmann M, Kessler B, La Thangue NB. Arginine methylation regulates the p53 response. *Nat Cell Biol* (2008) **10**:1431–1439. doi: 10.1038/ncb1802

52. Guo Z, Zheng L, Xu H, Dai H, Zhou M, Pascua MR, Chen QM, Shen B. Methylation of FEN1 suppresses nearby phosphorylation and facilitates PCNA binding. *Nat Chem Biol* (2010) **6**:766–773. doi: 10.1038/nchembio.422

53. Lu X, Fernando TM, Lossos C, Yusufova N, Liu F, Fontán L, Durant M, Geng H, Melnick J, Luo Y, et al. PRMT5 interacts with the BCL6 oncoprotein and is required for germinal center formation and lymphoma cell survival. *Blood* (2018) **132**:2026–2039. doi: 10.1182/blood-2018-02-831438

54. Teng Y, Girvan AC, Casson LK, Pierce WM, Qian M, Thomas SD, Bates PJ. AS1411 alters the localization of a complex containing protein arginine methyltransferase 5 and nucleolin. *Cancer Res* (2007) **67**:10491–10500. doi: 10.1158/0008-5472.CAN-06-4206

55. Hsu J-M, Chen C-T, Chou C-K, Kuo H-P, Li L-Y, Lin C-Y, Lee H-J, Wang Y-N, Liu M, Liao H-W, et al. Crosstalk between Arg 1175 methylation and Tyr 1173 phosphorylation negatively modulates EGFR-mediated ERK activation. *Nat Cell Biol* (2011) **13**:174–181. doi: 10.1038/ncb2158

56. Ma D, Yang M, Wang Q, Sun C, Shi H, Jing W, Bi Y, Shen X, Ma X, Qin Z, et al. Arginine methyltransferase PRMT5 negatively regulates cGAS-mediated antiviral immune response. *Sci Adv* (2021) **7**:eabc1834. doi: 10.1126/sciadv.abc1834

57. Rengasamy M, Zhang F, Vashisht A, Song W-M, Aguilo F, Sun Y, Li S, Zhang W, Zhang B, Wohlschlegel JA, et al. The PRMT5/WDR77 complex regulates alternative splicing through ZNF326 in breast cancer. *Nucleic Acids Res* (2017) **45**:11106–11120. doi: 10.1093/nar/gkx727

58. Gao G, Dhar S, Bedford MT. PRMT5 regulates IRES-dependent translation via methylation of hnRNP A1. *Nucleic Acids Res* (2017) **45**:4359–4369. doi: 10.1093/nar/gkw1367

59. Andreu-Pérez P, Esteve-Puig R, de Torre-Minguela C, López-Fauqued M, Bech-Serra JJ, Tenbaum S, García-Trevijano ER, Canals F, Merlino G, Avila MA, et al. Protein arginine methyltransferase 5 regulates ERK1/2 signal transduction amplitude and cell fate through CRAF. *Sci Signal* (2011) **4**:ra58. doi: 10.1126/scisignal.2001936

60. Hwang JW, Kim S-N, Myung N, Song D, Han G, Bae G-U, Bedford MT, Kim YK. PRMT5 promotes DNA repair through methylation of 53BP1 and is regulated by Src-mediated phosphorylation. *Commun Biol* (2020) **3**:428. doi: 10.1038/s42003-020-01157-z

61. He W, Ma X, Yang X, Zhao Y, Qiu J, Hang H. A role for the arginine methylation of Rad9 in checkpoint control and cellular sensitivity to DNA damage. *Nucleic Acids Res* (2011) **39**:4719–4727. doi: 10.1093/nar/gkq1264

62. Zheng S, Moehlenbrink J, Lu Y-C, Zalmas L-P, Sagum CA, Carr S, McGouran JF, Alexander L, Fedorov O, Munro S, et al. Arginine methylation-dependent reader-writer interplay governs growth control by E2F-1. *Mol Cell* (2013) **52**:37–51. doi: 10.1016/j.molcel.2013.08.039

63. Hu D, Gur M, Zhou Z, Gamper A, Hung M-C, Fujita N, Lan L, Bahar I, Wan Y. Interplay between arginine methylation and ubiquitylation regulates KLF4-mediated genome stability and carcinogenesis. *Nat Commun* (2015) **6**:8419. doi: 10.1038/ncomms9419

64. Clarke TL, Sanchez-Bailon MP, Chiang K, Reynolds JJ, Herrero-Ruiz J, Bandeiras TM, Matias PM, Maslen SL, Skehel JM, Stewart GS, et al. PRMT5-Dependent Methylation of the TIP60 Coactivator RUVBL1 Is a Key Regulator of Homologous Recombination. *Mol Cell* (2017) **65**:900-916.e7. doi: 10.1016/j.molcel.2017.01.019

65. Hyllus D, Stein C, Schnabel K, Schiltz E, Imhof A, Dou Y, Hsieh J, Bauer U-M. PRMT6-mediated methylation of R2 in histone H3 antagonizes H3 K4 trimethylation. *Genes Dev* (2007) **21**:3369–3380. doi: 10.1101/gad.447007

66. Kim S, Kim NH, Park JE, Hwang JW, Myung N, Hwang K-T, Kim YA, Jang C-Y, Kim YK. PRMT6-mediated H3R2me2a guides Aurora B to chromosome arms for proper chromosome segregation. *Nat Commun* (2020) **11**:612. doi: 10.1038/s41467-020-14511-w

67. Chan LH, Zhou L, Ng KY, Wong TL, Lee TK, Sharma R, Loong JH, Ching YP, Yuan Y-F, Xie D, et al. PRMT6 Regulates RAS/RAF Binding and MEK/ERK-Mediated Cancer Stemness Activities in Hepatocellular Carcinoma through CRAF Methylation. *Cell Rep* (2018) **25**:690-701.e8. doi: 10.1016/j.celrep.2018.09.053

68. Huang T, Yang Y, Song X, Wan X, Wu B, Sastry N, Horbinski CM, Zeng C, Tiek D, Goenka A, et al. PRMT6 methylation of RCC1 regulates mitosis, tumorigenicity, and radiation response of glioblastoma stem cells. *Mol Cell* (2021) **81**:1276-1291.e9. doi: 10.1016/j.molcel.2021.01.015

69. Choi S, Jeong H-J, Kim H, Choi D, Cho S-C, Seong JK, Koo S-H, Kang J-S. Skeletal muscle-specific Prmt1 deletion causes muscle atrophy via deregulation of the PRMT6-FOXO3 axis. *Autophagy* (2019) **15**:1069–1081. doi: 10.1080/15548627.2019.1569931

70. Che N, Ng K-Y, Wong T-L, Tong M, Kau PW, Chan L-H, Lee TK, Huen MS, Yun J-P, Ma S. PRMT6 deficiency induces autophagy in hostile microenvironments of hepatocellular carcinoma tumors by regulating BAG5-associated HSC70 stability. *Cancer Lett* (2021) **501**:247–262. doi: 10.1016/j.canlet.2020.11.002

71. Zhang H, Han C, Li T, Li N, Cao X. The methyltransferase PRMT6 attenuates antiviral innate immunity by blocking TBK1-IRF3 signaling. *Cell Mol Immunol* (2019) **16**:800–809. doi: 10.1038/s41423-018-0057-4

72. Lee S-H, Chen T-Y, Dhar SS, Gu B, Chen K, Kim YZ, Li W, Lee MG. A feedback loop comprising PRMT7 and miR-24-2 interplays with Oct4, Nanog, Klf4 and c-Myc to regulate stemness. *Nucleic Acids Res* (2016) **44**:10603–10618. doi: 10.1093/nar/gkw788

73. Jeong H-J, Lee H-J, Vuong TA, Choi K-S, Choi D, Koo S-H, Cho SC, Cho H, Kang J-S. Prmt7 Deficiency Causes Reduced Skeletal Muscle Oxidative Metabolism and Age-Related Obesity. *Diabetes* (2016) **65**:1868–1882. doi: 10.2337/db15-1500

74. Liu L, Zhang X, Ding H, Liu X, Cao D, Liu Y, Liu J, Lin C, Zhang N, Wang G, et al. Arginine and lysine methylation of MRPS23 promotes breast cancer metastasis through regulating OXPHOS. *Oncogene* (2021) **40**:3548–3563. doi: 10.1038/s41388-021-01785-7

75. Yao R, Jiang H, Ma Y, Wang L, Wang L, Du J, Hou P, Gao Y, Zhao L, Wang G, et al. PRMT7 induces epithelial-to-mesenchymal transition and promotes metastasis in breast cancer. *Cancer Res* (2014) **74**:5656–5667. doi: 10.1158/0008-5472.CAN-14-0800

76. Yang Y, Hadjikyriacou A, Xia Z, Gayatri S, Kim D, Zurita-Lopez C, Kelly R, Guo A, Li W, Clarke SG, et al. PRMT9 is a type II methyltransferase that methylates the splicing factor SAP145. *Nat Commun* (2015) **6**:6428. doi: 10.1038/ncomms7428
