# Supplementary material for: Protein Arginine Methylation: An Emerging Modification in Cancer Immunity and Immunotherapy
Source: Front Immunol. 2022 Apr 14;13:865964. doi: 10.3389/fimmu.2022.865964 (PMC9046588; doi:10.3389/fimmu.2022.865964)
Supplement: Supplementary file 2 [file Table_2.docx]

**Supplementary Table 2**

The biological mechanism of PRMTs in cancer.

| **PRMT** | **Cancer Types** | **Substrates** | **Biological function** | **Ref** |
| --- | --- | --- | --- | --- |
| PRMT1 | breast cancer | ERα | Enhance ERα and IGF-1 signaling | (1) |
|  |  | C/EBPα | Promote the expression of cyclin D1 | (2) |
|  |  | EZH2 | Enhance EMT | (3) |
|  | Pancreatic cancer | GLI1 | Enhance transcriptional activity of GLI1 | (4) |
|  | lung cancer | Twist1 | Enhance EMT | (5) |
|  | colorectal cancer | NONO | Enhance oncogenic function of NONO | (6) |
|  |  | SMARCA4 | Enhance EGFR signaling | (7) |
|  | leukaemia | FLT3 | Enhance the maintenance of FLT3-ITD+ AML | (8) |
| PRMT2 | breast cancer | ERα | Enhance ERα signaling | (9) |
|  | hepatocellular carcinoma | *Bcl2* | Promote the expression of *Bcl2* | (10) |
|  | glioblastoma | H3R8 | Activate oncogenic transcription | (11) |
| PRMT3 | colorectal cancer | HIF1α | Enhance the stabilization and oncogenic function of HIF1α | (12) |
|  | pancreatic cancer | GAPDH | Enhance glycolysis and mitochondrial respiration | (13) |
| CARM1 | breast cancer | LSD1 | Repress E-cadherin and activate vimentin transcription | (14) |
|  |  | ERα | Activate ERα-target genes transcription | (15) |
|  | ovarian cancer | BAF155 | Silence EZH2/BAF155 target tumor suppressor genes | (16) |
|  | liver cancer | GAPDH | Represses glycolytic flux and glycolysis | (17) |
|  | pancreatic cancer | MDH1 | Represses mitochondrial respiration and glutamine metabolism | (18) |
| PRMT5 | breast cancer | ZNF326 | Enhance alternative splicing of target genes | (19) |
|  |  | FOXP1 | Promote the expression of FOXP1 | (20) |
|  | lung cancer | miR-99 family | Activate the FGFR3/ERK/AKT signaling pathway | (21) |
|  |  | AKT | Enhance AKT activity | (22) |
|  | gastric cancer | c-Myc | Promote c-Myc-target genes transcription | (23) |
|  | prostate cancer | AR | Activate AR transcription | (24) |
|  | leukaemia | RB family | Silence *Rb1* gene | (25) |
| PRMT6 | lung cancer | ILF2 | Promote the alternate activation of TAM | (26) |
|  | gastric cancer | PCDH7, SCD and IGFBP5 | Repress tumor suppressor genes | (27) |
| PRMT7 | breast cancer | E-cadherin | Enhance EMT | (28) |
|  | renal cell carcinoma | β-catenin | Promote the expression of c-Myc | (29) |
| PRMT8 | colon cancer | *Sox2*, *Nanog* and *Oct4* | Enhances the cancer stem cell function | (30) |
| PRMT9 | hepatocellular carcinoma | PI3K/AKT/GSK-3β/Snail signaling pathway | Enhance EMT | (31) |

**References**

1. Choucair A, Pham TH, Omarjee S, Jacquemetton J, Kassem L, Trédan O, Rambaud J, Marangoni E, Corbo L, Treilleux I, et al. The arginine methyltransferase PRMT1 regulates IGF-1 signaling in breast cancer. *Oncogene* (2019) **38**:4015–4027. doi: 10.1038/s41388-019-0694-9

2. Liu L-M, Sun W-Z, Fan X-Z, Xu Y-L, Cheng M-B, Zhang Y. Methylation of C/EBPα by PRMT1 Inhibits Its Tumor-Suppressive Function in Breast Cancer. *Cancer Res* (2019) **79**:2865–2877. doi: 10.1158/0008-5472.CAN-18-3211

3. Li Z, Wang D, Lu J, Huang B, Wang Y, Dong M, Fan D, Li H, Gao Y, Hou P, et al. Methylation of EZH2 by PRMT1 regulates its stability and promotes breast cancer metastasis. *Cell Death Differ* (2020) **27**:3226–3242. doi: 10.1038/s41418-020-00615-9

4. Wang Y, Hsu J-M, Kang Y, Wei Y, Lee P-C, Chang S-J, Hsu Y-H, Hsu JL, Wang H-L, Chang W-C, et al. Oncogenic Functions of Gli1 in Pancreatic Adenocarcinoma Are Supported by Its PRMT1-Mediated Methylation. *Cancer Res* (2016) **76**:7049–7058. doi: 10.1158/0008-5472.CAN-16-0715

5. Avasarala S, Van Scoyk M, Karuppusamy Rathinam MK, Zerayesus S, Zhao X, Zhang W, Pergande MR, Borgia JA, DeGregori J, Port JD, et al. PRMT1 Is a Novel Regulator of Epithelial-Mesenchymal-Transition in Non-small Cell Lung Cancer. *J Biol Chem* (2015) **290**:13479–13489. doi: 10.1074/jbc.M114.636050

6. Yin X-K, Wang Y-L, Wang F, Feng W-X, Bai S-M, Zhao W-W, Feng L-L, Wei M-B, Qin C-L, Wang F, et al. PRMT1 enhances oncogenic arginine methylation of NONO in colorectal cancer. *Oncogene* (2021) **40**:1375–1389. doi: 10.1038/s41388-020-01617-0

7. Yao B, Gui T, Zeng X, Deng Y, Wang Z, Wang Y, Yang D, Li Q, Xu P, Hu R, et al. PRMT1-mediated H4R3me2a recruits SMARCA4 to promote colorectal cancer progression by enhancing EGFR signaling. *Genome Med* (2021) **13**:58. doi: 10.1186/s13073-021-00871-5

8. He X, Zhu Y, Lin Y-C, Li M, Du J, Dong H, Sun J, Zhu L, Wang H, Ding Z, et al. PRMT1-mediated FLT3 arginine methylation promotes maintenance of FLT3-ITD+ acute myeloid leukemia. *Blood* (2019) **134**:548–560. doi: 10.1182/blood.2019001282

9. Zhong J, Cao R-X, Zu X-Y, Hong T, Yang J, Liu L, Xiao X-H, Ding W-J, Zhao Q, Liu J-H, et al. Identification and characterization of novel spliced variants of PRMT2 in breast carcinoma. *FEBS J* (2012) **279**:316–335. doi: 10.1111/j.1742-4658.2011.08426.x

10. Hu G, Yan C, Xie P, Cao Y, Shao J, Ge J. PRMT2 accelerates tumorigenesis of hepatocellular carcinoma by activating Bcl2 via histone H3R8 methylation. *Exp Cell Res* (2020) **394**:112152. doi: 10.1016/j.yexcr.2020.112152

11. Dong F, Li Q, Yang C, Huo D, Wang X, Ai C, Kong Y, Sun X, Wang W, Zhou Y, et al. PRMT2 links histone H3R8 asymmetric dimethylation to oncogenic activation and tumorigenesis of glioblastoma. *Nat Commun* (2018) **9**:4552. doi: 10.1038/s41467-018-06968-7

12. X Z, K W, X F, J W, Y C, C J, Q H, C C. PRMT3 promotes tumorigenesis by methylating and stabilizing HIF1α in colorectal cancer. *Cell Death Dis* (2021) **12**: doi: 10.1038/s41419-021-04352-w

13. Hsu M-C, Tsai Y-L, Lin C-H, Pan M-R, Shan Y-S, Cheng T-Y, Cheng SH-C, Chen L-T, Hung W-C. Protein arginine methyltransferase 3-induced metabolic reprogramming is a vulnerable target of pancreatic cancer. *J Hematol OncolJ Hematol Oncol* (2019) **12**:79. doi: 10.1186/s13045-019-0769-7

14. Liu J, Feng J, Li L, Lin L, Ji J, Lin C, Liu L, Zhang N, Duan D, Li Z, et al. Arginine methylation-dependent LSD1 stability promotes invasion and metastasis of breast cancer. *EMBO Rep* (2020) **21**:e48597. doi: 10.15252/embr.201948597

15. Peng B-L, Li W-J, Ding J-C, He Y-H, Ran T, Xie B-L, Wang Z-R, Shen H-F, Xiao R-Q, Gao W-W, et al. A hypermethylation strategy utilized by enhancer-bound CARM1 to promote estrogen receptor α-dependent transcriptional activation and breast carcinogenesis. *Theranostics* (2020) **10**:3451–3473. doi: 10.7150/thno.39241

16. Karakashev S, Zhu H, Wu S, Yokoyama Y, Bitler BG, Park P-H, Lee J-H, Kossenkov AV, Gaonkar KS, Yan H, et al. CARM1-expressing ovarian cancer depends on the histone methyltransferase EZH2 activity. *Nat Commun* (2018) **9**:631. doi: 10.1038/s41467-018-03031-3

17. Zhong X-Y, Yuan X-M, Xu Y-Y, Yin M, Yan W-W, Zou S-W, Wei L-M, Lu H-J, Wang Y-P, Lei Q-Y. CARM1 Methylates GAPDH to Regulate Glucose Metabolism and Is Suppressed in Liver Cancer. *Cell Rep* (2018) **24**:3207–3223. doi: 10.1016/j.celrep.2018.08.066

18. Wang Y-P, Zhou W, Wang J, Huang X, Zuo Y, Wang T-S, Gao X, Xu Y-Y, Zou S-W, Liu Y-B, et al. Arginine Methylation of MDH1 by CARM1 Inhibits Glutamine Metabolism and Suppresses Pancreatic Cancer. *Mol Cell* (2016) **64**:673–687. doi: 10.1016/j.molcel.2016.09.028

19. Rengasamy M, Zhang F, Vashisht A, Song W-M, Aguilo F, Sun Y, Li S, Zhang W, Zhang B, Wohlschlegel JA, et al. The PRMT5/WDR77 complex regulates alternative splicing through ZNF326 in breast cancer. *Nucleic Acids Res* (2017) **45**:11106–11120. doi: 10.1093/nar/gkx727

20. Chiang K, Zielinska AE, Shaaban AM, Sanchez-Bailon MP, Jarrold J, Clarke TL, Zhang J, Francis A, Jones LJ, Smith S, et al. PRMT5 Is a Critical Regulator of Breast Cancer Stem Cell Function via Histone Methylation and FOXP1 Expression. *Cell Rep* (2017) **21**:3498–3513. doi: 10.1016/j.celrep.2017.11.096

21. Jing P, Zhao N, Ye M, Zhang Y, Zhang Z, Sun J, Wang Z, Zhang J, Gu Z. Protein arginine methyltransferase 5 promotes lung cancer metastasis via the epigenetic regulation of miR-99 family/FGFR3 signaling. *Cancer Lett* (2018) **427**:38–48. doi: 10.1016/j.canlet.2018.04.019

22. Zhang S, Ma Y, Hu X, Zheng Y, Chen X. Targeting PRMT5/Akt signalling axis prevents human lung cancer cell growth. *J Cell Mol Med* (2019) **23**:1333–1342. doi: 10.1111/jcmm.14036

23. Liu M, Yao B, Gui T, Guo C, Wu X, Li J, Ma L, Deng Y, Xu P, Wang Y, et al. PRMT5-dependent transcriptional repression of c-Myc target genes promotes gastric cancer progression. *Theranostics* (2020) **10**:4437–4452. doi: 10.7150/thno.42047

24. Beketova E, Fang S, Owens JL, Liu S, Chen X, Zhang Q, Asberry AM, Deng X, Malola J, Huang J, et al. Protein Arginine Methyltransferase 5 Promotes pICln-Dependent Androgen Receptor Transcription in Castration-Resistant Prostate Cancer. *Cancer Res* (2020) **80**:4904–4917. doi: 10.1158/0008-5472.CAN-20-1228

25. Wang L, Pal S, Sif S. Protein arginine methyltransferase 5 suppresses the transcription of the RB family of tumor suppressors in leukemia and lymphoma cells. *Mol Cell Biol* (2008) **28**:6262–6277. doi: 10.1128/MCB.00923-08

26. Avasarala S, Wu P-Y, Khan SQ, Yanlin S, Van Scoyk M, Bao J, Di Lorenzo A, David O, Bedford MT, Gupta V, et al. PRMT6 Promotes Lung Tumor Progression via the Alternate Activation of Tumor-Associated Macrophages. *Mol Cancer Res MCR* (2020) **18**:166–178. doi: 10.1158/1541-7786.MCR-19-0204

27. Okuno K, Akiyama Y, Shimada S, Nakagawa M, Tanioka T, Inokuchi M, Yamaoka S, Kojima K, Tanaka S. Asymmetric dimethylation at histone H3 arginine 2 by PRMT6 in gastric cancer progression. *Carcinogenesis* (2019) **40**:15–26. doi: 10.1093/carcin/bgy147

28. Yao R, Jiang H, Ma Y, Wang L, Wang L, Du J, Hou P, Gao Y, Zhao L, Wang G, et al. PRMT7 induces epithelial-to-mesenchymal transition and promotes metastasis in breast cancer. *Cancer Res* (2014) **74**:5656–5667. doi: 10.1158/0008-5472.CAN-14-0800

29. Liu F, Wan L, Zou H, Pan Z, Zhou W, Lu X. PRMT7 promotes the growth of renal cell carcinoma through modulating the β-catenin/C-MYC axis. *Int J Biochem Cell Biol* (2020) **120**:105686. doi: 10.1016/j.biocel.2020.105686

30. Lin H, Wang B, Yu J, Wang J, Li Q, Cao B. Protein arginine methyltransferase 8 gene enhances the colon cancer stem cell (CSC) function by upregulating the pluripotency transcription factor. *J Cancer* (2018) **9**:1394–1402. doi: 10.7150/jca.23835

31. Jiang H, Zhou Z, Jin S, Xu K, Zhang H, Xu J, Sun Q, Wang J, Xu J. PRMT9 promotes hepatocellular carcinoma invasion and metastasis via activating PI3K/Akt/GSK-3β/Snail signaling. *Cancer Sci* (2018) **109**:1414–1427. doi: 10.1111/cas.13598
